# Color-tunable ultralong organic room temperature phosphorescence from a multicomponent copolymer

**DOI:** 10.1038/s41467-020-14792-1

**Published:** 2020-02-18

**Authors:** Long Gu, Hongwei Wu, Huili Ma, Wenpeng Ye, Wenyong Jia, He Wang, Hongzhong Chen, Nan Zhang, Dongdong Wang, Cheng Qian, Zhongfu An, Wei Huang, Yanli Zhao

**Affiliations:** 1 Division of Chemistry and Biological Chemistry, School of Physical and Mathematical Sciences, Nanyang Technological University, 21 Nanyang Link, Singapore, 637371 Singapore; 2 Key Laboratory of Flexible Electronics & Institute of Advanced Materials, Jiangsu National Synergistic Innovation Center for Advanced Materials, Nanjing Tech University, 30 South Puzhu Road, Nanjing, 211816 P. R. China; 3 Shaanxi Institute of Flexible Electronics, Northwestern Polytechnical University, 127 West Youyi Road, Xi’an, 710072 P. R. China

**Keywords:** Self-assembly, Supramolecular polymers, Polymers

## Abstract

Functional materials displaying tunable emission and long-lived luminescence have recently emerged as a powerful tool for applications in information encryption, organic electronics and bioelectronics. Herein, we present a design strategy to achieve color-tunable ultralong organic room temperature phosphorescence (UOP) in polymers through radical multicomponent cross-linked copolymerization. Our experiments reveal that by changing the excitation wavelength from 254 to 370 nm, these polymers display multicolor luminescence spanning from blue to yellow with a long-lived lifetime of 1.2 s and a maximum phosphorescence quantum yield of 37.5% under ambient conditions. Moreover, we explore the application of these polymers in multilevel information encryption based on the color-tunable UOP property. This strategy paves the way for the development of multicolor bio-labels and smart luminescent materials with long-lived emission at room temperature.

## Abstract (web summary)

Functional materials displaying tunable emission and long-lived luminescence are a powerful tool in information encryption, organic electronics and bioelectronics. Here the authors design a color-tunable ultralong organic room temperature phosphorescence polymer through radical multiple component cross-linked copolymerization.

## Introduction

Room-temperature phosphorescence with ultralong emission lifetime has garnered tremendous interests in recent years due to their unique long-lived and rich excited-state features^1–4^, which can be used in a wide array of potential technological applications, spanning from decorative and emergency displays to information anti-counterfeiting and bio-applications^5–10^. Nevertheless, this fascinating luminescence phenomenon was considered an exclusive property of inorganic materials in the past decades on account of the inefficient intersystem crossing (ISC) and intensive non-radiative decay of organic luminophores under ambient conditions. The intrinsic disadvantages of these inorganic counterparts such as the harsh preparation conditions, the scarcity of metal resource and high toxicity for bio-application have led to the search for metal-free alternatives^11^. In the recent years, alternative molecule design rules and enhanced strategies such as crystal engineering^12–16^, host–guest doping^17^, metal–organic frameworks (MOFs)^18^, H-aggregation^19,20^, and others^21–23^ are proposed to achieve ultralong organic room-temperature phosphorescence (UOP) through improving the ISC and suppressing the non-radiation decay of triplet excitons. Generally, constructing a rigid environment through crystal engineering is the most common approach to obtain the long-lived phosphorescence emission at room temperature^24^. However, the crystal-based UOP materials have problems of reproducibility, processability, and flexibility, which greatly hinder the development of crystal-based UOP materials for practical applications^25,26^. To overcome these fundamental hurdles, special attention has been paid to the development of organic polymeric materials capable of emitting ultralong phosphorescence at room temperature. Although significant breakthroughs have been achieved by prolonging the lifetime of UOP based on polymeric materials through the construction of a rigid polymer microenvironment via homopolymerization^27^ and radical binary copolymerization^28^, as well as embedding small molecules into a rigid polymer matrix^29^, research on color tunability of polymer-based UOP in a single polymer under ambient conditions has not been reported.

Color-tunable luminescent materials have gained considerable attention owing to their potential applications in multicolor display, polychromatic imaging agents in biological applications, information encryption, and anti-counterfeiting^30–33^. Various strategies have been developed to create materials with color-tunable emission in both small molecule and polymeric systems, which include modulating the composition of materials, changing the molecular conformations, and regulating the molecular packing mode in crystals^34–39^. Despite the successful developments of fluorescence materials with multicolor emission by rational molecular design and engineering, it is still a great challenge to obtain room-temperature phosphorescence materials that demonstrate color tunability in response to an external stimulus, such as light, electric fields, humidity, and pressure.

**Fig. 1:**
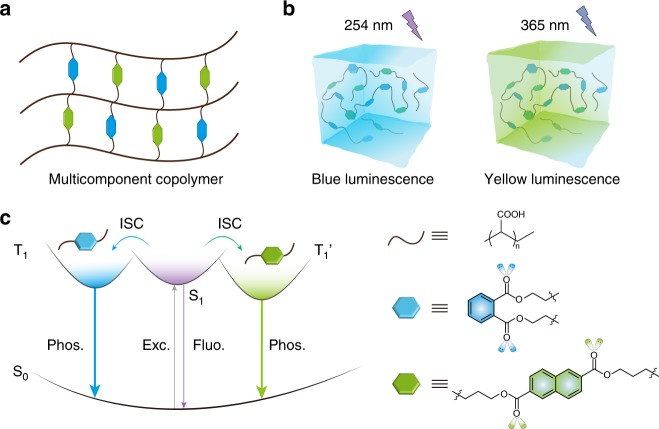
Schematic illustration of the color-tunable UOP multicomponent copolymer. **a** Network structure of multicomponent copolymer by radical cross-linking copolymerization. The brown line and blue and yellow hexagons represent PAA chains and various luminophores, respectively. **b** Excitation-dependent UOP of multi-component copolymer under ambient conditions. The polymer exhibits color-tunable UOP emission upon changes in the excitation wavelength. **c** Proposed mechanism of color-tunable UOP at room temperature. Multiple triplet excited state T_1_ and T_1_’ generating from different excited singlet states *S*_1_ stem from the various luminophores through ISC. The rigid polymer microenvironment effectively restricts molecular motion for ultralong phosphorescence emission under ambient conditions.

Inspired by the common methods of achieving multicolor emission in organic light-emitting diodes through the incorporation of luminophores with different emission colors into one polymer as well as the construction of multiple emitting centers in a single-component molecular crystal to obtain multicolor room-temperature phosphorescence emission under different excitation wavelengths, we propose that excitation wavelength-responsive UOP may be obtained by conjugating multiple UOP emitting centers onto a polymer backbone through radical cross-linked copolymerization under ambient conditions (Fig. 1a, b).

## Results

### Photophysical properties of PDNA polymer

To validate our hypothesis, we designed and prepared a multicomponent copolymer by radical cross-linked copolymerization of acrylic acid and multiple luminophores (Fig. 1c). Polyacrylic acid (PAA) contains numerous carbonyl and hydroxyl groups, which not only enhance spin–orbit coupling by improving the ISC from singlet-to-triplet excited states for generating the triplet excitons but also restrict molecular motion by constructing an intermolecular/intramolecular hydrogen bonding network between the polymer matrix and luminophores, suppressing the non-radiative decay of excited triplet state. In order to achieve the color-tunable UOP emission under different excitation wavelengths, chromophores with different degrees of conjugation were incorporated into the PAA chains though functionalized alkyl chains, which may avoid undesirable complications caused by energy transfer or aggregation. Meanwhile, the formation of a cross-linked polymer network may further enhance the rigidity of polymer matrix for generating long-lived phosphorescence emission under ambient conditions.

As a proof of concept, we synthesized a copolymer (PDNA) by radical cross-linked copolymerization of acrylic acid (AA), vinyl-functionalized naphthalene (MND), and benzene (MDP) using 2-azoisobutyronitrile (AIBN) as an initiator (Supplementary Methods and Supplementary Figs. 1–3). The chemical structures were characterized by nuclear magnetic resonance (NMR) spectroscopy (Supplementary Figs. 4–10). The molar feed ratio of MND/MDP/AA was 1/200/10,000. The polymer PDNA shows number-average molecular weights (Mn) of 67.3 K Da (Supplementary Table 1). As anticipated, blue long-lived luminescence of approximately 4 s was observed by the naked eye from the transparent polymer film PDNA after switching off the ultraviolet (UV) lamp of 254 nm under ambient conditions (Supplementary Fig. 11 and Supplementary Movie 1). Notably, when the excitation wavelength was changed from 254 to 365 nm, a distinctly different yellow color long-lived emission that lasted for approximately 9 s could be observed after switching off the UV lamp, exhibiting an excitation wavelength-responsive ultralong luminescence characteristic.

**Fig. 2:**
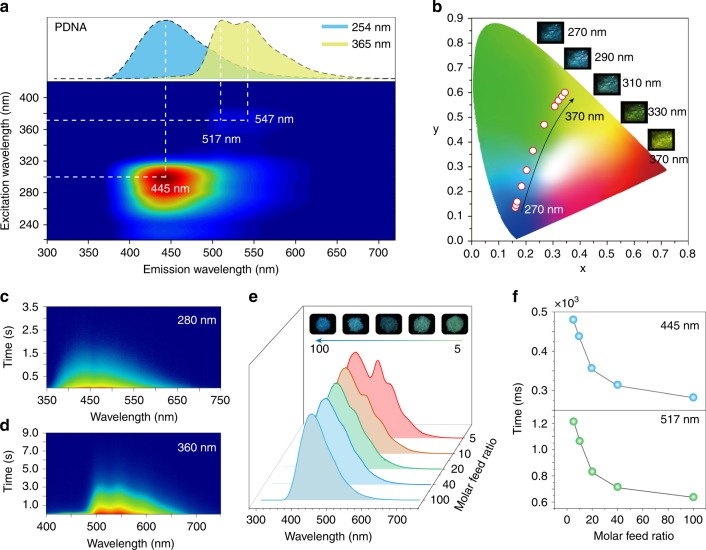
Photophysical properties of multicomponent copolymer at room temperature. **a** Excitation–phosphorescence mapping of polymer film PDNA under ambient conditions. The inset displays the phosphorescence spectra of the transparent film recorded at room temperature excited by 254 nm (blue) and 365 nm (yellow). **b** CIE chromaticity diagram for polymer PDNA with excitation varied from 270 to 370 nm. Inset: long-lived luminescence photographs of polymer film PDNA excited at 270, 290, 310, 330 and 370 nm, respectively. **c**, **d** Time-resolved emission spectra of PDNA at room temperature upon excitation at 280 and 360 nm, respectively. **e** Normalized phosphorescence spectra and the ultralong phosphorescence photographs (inserted images) of multicomponent copolymers with different molar feed ratio of MND:MDP:AA = 1:5:1000 (PDNA-5), 1:10:1000 (PDNA-10), 1:20:1000 (PDNA-20), 1:40:1000 (PDNA-40), and 1:100:1000 (PDNA-100) excited at 254 nm. **f** Phosphorescence lifetime of PDNA with different molar feed ratios of PDNA-5, PDNA-10, PDNA-20, PDNA-40, and PDNA-100 at 445 (top) and 517 nm (bottom) when excited at 254 and 360 nm at room temperature, respectively.

To further investigate on this interesting color-tunable long-lived luminescence phenomenon, we performed the excitation–phosphorescence spectra measurements of the PDNA polymer film at room temperature. As illustrated in Fig. 2a, when the excitation wavelength varied from 220 to 420 nm, the main emission center of polymer PDNA showed a significant bathochromic shift from 445 to 547 nm, accompanied by the long-lived luminescence color turned from blue to yellow under ambient conditions (Supplementary Fig. 12). Furthermore, Commission International de l’Eclairage (CIE) coordinate diagram of the PDNA film was calculated from their phosphorescence spectra excited at corresponding wavelengths to demonstrate the color change of PDNA (Fig. 2b). As the excitation wavelength increased from 270 to 370 nm, the variation in long-lived luminescence color spanned from blue to yellow, demonstrating good linearity on the CIE coordinate. In addition, similar color-tunable long-lived luminescence phenomenon for polymer PDNA can also be observed at low temperature (77 K) and oxygen atmosphere (Supplementary Figs. 13 and 14), indicating that the external environment has little influence on the performance of color-tunable long-lived emission. Meanwhile, the steady-state photoluminescence spectra of polymer PDNA also exhibit an excitation wavelength-responsive luminescence property under ambient conditions (Supplementary Fig. 15).

Analysis of the time-resolved phosphorescence emission spectra of polymer PDNA at room temperature provided insights on its long-lived luminescence property. As shown in Fig. 2c, d, the main emission bands at 445, 517, and 547 nm in PDNA show an ultralong lifetime of 428, 949, and 1096 ms with a phosphorescence quantum yield of 23.2% upon excitation at 280 and 360 nm, respectively (Supplementary Fig. 16 and Supplementary Table 2), indicating phosphorescence emission characteristics. This phenomenon was further confirmed through oxygen-quenching phosphorescence experiments. Compared with the phosphorescence spectra of PDNA in nitrogen atmosphere, it was found that the intensity of ultralong emission peaks displayed a significant reduction under an oxygenated environment, which is the property of phosphorescence emission (Supplementary Fig. 17).

### Effect of different molar feed ratio on UOP

The ratio variation of different luminophores may have a great influence on the luminescence color in the polymer. Hence, we systematically explored the effects of the variation in molar feed ratio of MND/MDP/AA on the photophysical properties in the resulting copolymer. A series of multicomponent copolymers with different molar feed ratio ranging from 1:5:1000 to 1:100:1000 were prepared. As the molar feed ratio of MND/MDP/AA changed from 1:5:1000 (PDNA-5), 1:10:1000 (PDNA-10), 1:20:1000 (PDNA-20), 1:40:1000 (PDNA-40) to 1:100:1000 (PDNA-100), the phosphorescence intensity of bands at 517 and 547 nm decreased gradually under excitation at 254 nm, resulting in the hyperchromatic shift of long-lived luminescence color from green to blue under ambient conditions (Fig. 2e and Supplementary Fig. 18), displaying molar feed ratio dependent UOP property. Nevertheless, when the excitation wavelength was at 365 nm, these polymers all displayed long-lived yellow luminescence after switching off the excitation source (Supplementary Fig. 19). Moreover, when the molar ratio of MDP increased from 5 to 100, the lifetime of these polymers at 445 and 517 nm showed a significant decrease from 479.7 and 1222 ms to 282 and 636.2 ms, respectively (Fig. 2f and Supplementary Figs. 20–22 and Supplementary Table 3). This decease of lifetime in polymers might be assigned to the intensive non-radiative transition and quenching of triplet excitons by motion and collision of phosphors in polymer matrix, resulting from the incorporation of abundant luminophores into polymer chains. In addition, the polymer PDNA also displays time-dependent phosphorescence emission, with the delay time prolonged from 5 to 500 ms and the phosphorescence emission band at 445 nm decreased gradually, leading to the color change on the CIE chromaticity coordinates (Supplementary Fig. 23)

### Proposed mechanism for color-tunable UOP

**Fig. 3:**
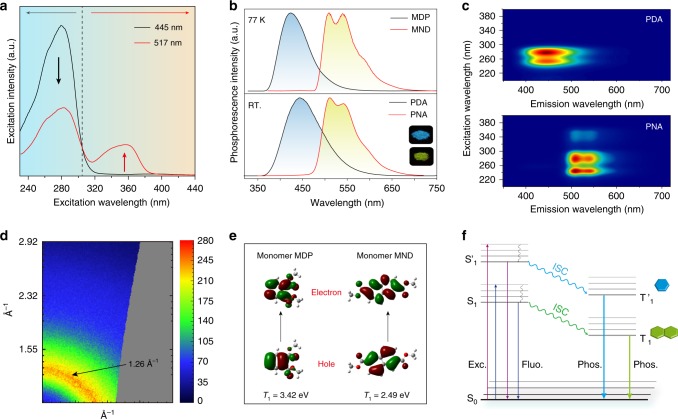
Mechanism of color-tunable UOP in multicomponent copolymer at room temperature. **a** Phosphorescence excitation spectra in polymer PDNA at 445 and 517 nm. **b** Normalized phosphorescence spectra of monomer MDP and MND in 2-methyltetrahydrofuran (1 × 10^−5^ M) at 77 K (top), as well as polymer PDA and PNA at room temperature (RT) (bottom) excited at 280 and 360 nm. **c** Polymer film excitation–phosphorescence mapping of PDA (top) and PNA (bottom) at room temperature. **d** WAXS pattern of polymer film PDNA. **e** Natural transition orbitals for the lowest triplet transitions of MDP and MND in gaseous state. **f** Proposed mechanism of the color-tunable UOP in multicomponent copolymer. Fluo. fluorescence, Phos. molecular phosphorescence, Exc. excitation.

To probe the mechanism underlying this color-tunable phosphorescence emission in multicomponent copolymer, the phosphorescence excitation spectra of polymer film PDNA at 445 and 517 nm were recorded at room temperature. As shown in Fig. 3a, significantly different excitation spectra at 445 and 517 nm were observed in polymer PDNA. When the excitation wavelength is in the range from 230 to 302 nm, blue long-lived luminescence is more intense than the yellow emission. In contrast, phosphorescence emission was dominated by the yellow luminescence when the excitation wavelength ranges from 303 to 390 nm. The ratiometric variation of phosphorescence emission intensity at 445, 517, and 547 nm upon changes in the excitation wavelength leads to color-tunable long-lived luminescence, which agrees with the excitation-dependent ultralong organic phosphorescence spectra. More importantly, from the analysis of the excitation spectra, it was revealed that the blue and yellow emission bands at 445 and 517 nm stem from two different excited triplet states. Considering that the two luminophores were introduced into the PAA chains, we thus speculated that the two triplet state emission may have originated from the monomer phosphorescence luminescence of vinyl-functionalized luminophores MDP and MND in this copolymer.

To verify this, the photophysical properties of monomer MDP and MND were investigated in both solid state and diluted solution (Supplementary Note 1 and Supplementary Fig. 24). In the solid state, the samples MDP and MND displayed intensive blue fluorescence emission at 367 and 382 nm as well as at 387 and 405 nm, respectively. However, no phosphorescence signal could be detected at room temperature (Supplementary Fig. 25). In addition, the phosphorescence properties of the monomers MDP and MND in dilute 2-methyltetrahydrofuran (m-THF) solution at 77 K were studied (Fig. 3b, top). It was found that the emission shape and major peaks of monomer MDA and MNA at 424, 505, and 537 nm under excitation at 280 and 360 nm in m-THF solution were similar to the blue and yellow phosphorescence emission at 445, 517, and 547 nm in the multicomponent copolymer PDNA under ambient conditions, respectively. This result indicated that the blue and yellow ultralong phosphorescence emission in PDNA was attributed to the single-molecule phosphorescence of derivatives of benzene and naphthalene. In order to accurately investigate the photophysical property of the monomers MDP and MND in polymer, we synthesized another two kinds of two-component copolymers PDA and PNA containing single luminophore, by radical cross-linked copolymerization of monomers MDP, MND, and AA with the molar feed ratio 1:1000, respectively, and their photophysical properties were studied by phosphorescence spectra and lifetime profiles at room temperature (Supplementary Note 2 and Supplementary Figs. 26–29). As expected, the phosphorescence bands at around 443, 510, and 543 nm excited at corresponding excitation wavelengths of 280 and 360 nm in the polymers PDA and PNA agreed with the blue and yellow emission in polymer PDNA at room temperature (Fig. 3b, bottom and Supplementary Figs. 30 and 31). These control polymers all exhibited a long-lived luminescence with the lifetimes of 463, 842, and 835 ms at the corresponding emission bands (Supplementary Table 4).

As control experiments, to eliminate the possibility that the color-tunable UOP emission in polymer was a result of various aggregation emissions of polymer chains upon changes in the excitation wavelength, we also performed the measurement of excitation–phosphorescence spectra on binary copolymers PDA and PNA (Fig. 3c). It was shown that, when the excitation wavelength was varied from 200 to 400 nm, the ultralong organic phosphorescence was dominated by the blue and yellow emission in polymers PDA and PNA ascribing to the isolate phosphorescence emission of MDP and MND, respectively. No excitation-dependent UOP behavior was observed. Therefore, the variation in the excitation wavelength does not result in various aggregated state phosphorescence emission in polymer chains but rather modulates the intensity ratio of emission peaks at 445, 517, and 547 nm, leading to the color-tunable UOP in polymer PDNA. This illustration was further confirmed by the wide-angle X-ray scattering of the polymer PDNA. As shown in Fig. 3d, except the two broad scattering bands at around 1.27 Å (16.76°) and 2.41 Å (35.60°) were observed, which can be attributed to the scattering bands of PAA (Supplementary Fig. 32)^40^, no other *π*–*π* interactions from the aggregated state can be detected. From the result of powder X-ray diffraction, only two small broad diffraction bands at around 17° and 36° were obtained, indicating the amorphous nature of PDNA (Supplementary Fig. 33). Furthermore, theoretical calculation of the polymer PDNA was conducted to verify the above speculation on color-tunable UOP (Fig. 3e), the lowest triplet state (T_1_) of monomers MDP and MND is located at 362 and 498 nm in gaseous state, respectively, which is similar with the experimental data of 445 and 517 nm.

Taken together, it was suggested that the color-tunable luminescence from blue to yellow was achieved in polymer PDNA by modulating the phosphorescence intensity ratio of blue emission of monomer MDP and yellow emission of monomer MND through variations in the excitation wavelengths (Fig. 3f). Meanwhile, numerous hydrogen bonds among the PAA chains form a rigid polymer microenvironment, which not only effectively restrict molecular motion to suppress the non-radiative decay of excited state but also prevent the quenching of triplet excitons by the surroundings (oxygen, moisture, etc.) for generating the ultralong lifetime phosphorescence emission. This mechanism can be further confirmed by an experiment regarding the effect of humidity on the lifetime of polymers (Supplementary Fig. 34 and Supplementary Table 5). When the polymer film PDNA was exposed to moist atmosphere (humidity is 86%), the lifetime of PDNA at 445 and 517 nm exhibits a significant decrease within 60 min, which may be attributed to the breakage of the hydrogen bonds among polymer chains by moisture. Furthermore, as a control, a multicomponent polymer was prepared by physically embedding the monomers MDP and MND into PAA with a molar ratio of 1:5:1000. The resulting polymer showed weak phosphorescence emission signals at 445 and 517 nm with a short lifetime, which further indicated that the covalent cross-linked network of the polymer played a critical role in color-tunable UOP (Supplementary Figs. 35 and 36).

### General strategy for achieving color-tunable UOP

**Fig. 4:**
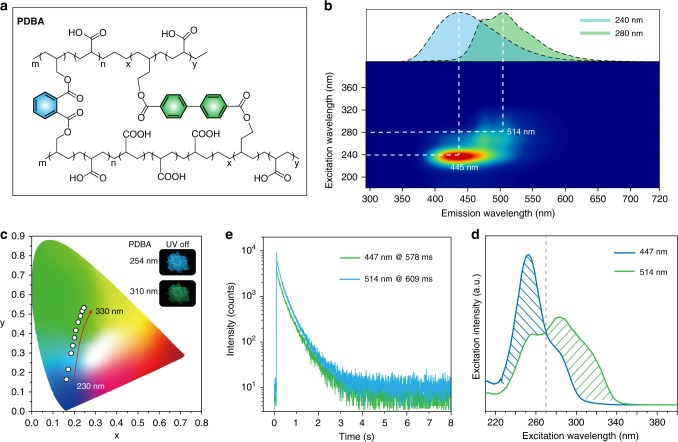
Molecular structure and phosphorescence properties of the multicomponent copolymer PDBA under ambient conditions. **a** Molecular structure of polymer PDBA. **b** Excitation–phosphorescence mapping of PDBA film. The inset image shows the phosphorescence spectra of PDBA excited by corresponding excitation wavelengths. **c** CIE chromaticity diagram for polymer PDBA with excitation varied from 230 to 330 nm. Inset: UOP photographs of polymer film PDBA excited at 254 and 310 nm after switching off the UV light, respectively. **d** Lifetime decay curves of bands at 447 and 514 nm in polymer PDBA, respectively. **e** Phosphorescence excitation spectra of polymer PDBA at 447 and 514 nm.

To establish the generality of our strategies for the generation of color-tunable UOP, another multicomponent copolymer PDBA was designed and prepared by radical cross-linked copolymerization of monomer di(but-3-en-1-yl) (1,1’-biphenyl)-4,4’-dicarboxylate (MBD), MDP, and AA with a molar feed ratio of 1:5:1000 (Fig. 4a and Supplementary Fig. 3). As expected, the polymer PDBA exhibited an excitation wavelength-dependent ultralong luminescence behavior under ambient conditions (Supplementary Movie 2). As shown in Fig. 4b, the emission bands of polymer PDBA displayed a gradual bathochromic shift from 445 to 514 nm when the excitation wavelength varied from 200 to 400 nm, demonstrating color-tunable emission characteristics (Supplementary Figs. 37 and 38). In addition, the ultralong emission color spans from blue to green with a long lifetime of 578 and 609 ms at 445 and 514 nm, respectively (Fig. 4c, e). Similar to PDNA, this color-tunable UOP property in the multicomponent copolymer PDBA is due to the dynamic ratio change of phosphorescence intensity from the different monomers MDP and MBD upon variations in the excitation wavelengths from 220 to 350 nm under ambient conditions (Fig. 4f and Supplementary Fig. 39).

### Potential application of color-tunable UOP

**Fig. 5:**
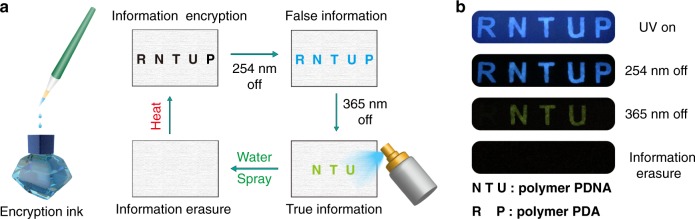
Description of color-tunable UOP for multilevel information encryption. **a** Process of information encryption by using the multi-component copolymer PDNA (NTU) and PDA (RP) as encryption ink under ambient conditions. **b** Long-lived luminescence photographs of letters (RNTUP and NTU) before and after switching off the UV light of 254 and 365 nm, respectively.

Given the excitation-dependent UOP emission property in the polymer PDNA, a potential application in multilevel information encryption was explored. As demonstrated in Fig. 5, the encryption information pattern “RNTUP” was fabricated by using the polymers PDA and PDNA as encryption inks, and the pattern was left to dry for 10 min at 75 °C in an oven. The letters “RP” and “NTU” show similar blue luminescence upon the UV irradiation at 254 nm. After switching off the UV light, the pattern “RNTUP” with blue long-lived luminescence was observed by the naked eyes under ambient conditions. The blue long-lived luminescence letters of “RNTUP” are false information. However, when the irradiation source changed from 254 to 365 nm, the true information of “NTU” with yellow long-lived phosphorescence emission can be captured after switching off the UV light. Therefore, using the excitation wavelength-responsive UOP nature of the polymer PDNA, multilevel information encryption can be achieved. Moreover, the information erasure function could be activated by spraying a small amount of water on the pattern. As shown in Fig. 5b, after spraying with water, the letters with phosphorescence luminescence signal disappeared when removed from the UV light of both 254 and 365 nm, indicating that moisture can weaken the hydrogen-bonding network between polymer chains and enhance the molecular motion in polymer chains, which in turn quench room-temperature phosphorescence. This process can be easily reversed by drying the pattern in the oven, exhibiting the reusability of such materials. Visible and multilevel information encryption is essential for the development of secure information storage and anti-counterfeiting.

## Discussion

In conclusion, we have presented an effective design strategy to achieve color-tunable UOP in single polymer through radical multicomponent cross-linked copolymerization. With the excitation wavelength changed from 254 to 370 nm, the UOP emission color of copolymer turned from blue (445 nm) to yellow (547 nm) with an extensive emission zone in the visible spectrum under ambient conditions. Based on the results of various experiments and theoretical calculation, the dynamic ratiometric variation in phosphorescence intensity of different luminophores is responsible for the color-tunable UOP. Rigid cross-linked polymer network and hydrogen bonding among polymer chains effectively suppress the motion of polymer chains and prevent the quenching of triplet excitons by oxygen and moisture, creating conditions necessary for generating long-lived phosphorescence at room temperature. Notably, these multicomponent copolymers exhibit a lifetime of 1.2 s and the maximum phosphorescence quantum yield of 37.5% under ambient conditions. This study not only paves a fresh way to the design and preparation of color-tunable UOP polymeric luminescence materials but also offers a guideline for developing materials toward multilevel information encryption, multicolor display, and biological applications.

## Methods

### Preparation of monomers and polymers

*Diallyl phthalate (MDP)*: Phthalic acid (0.50 g; 3.01 mmol), 4-(dimethylamino)pyridine (DMAP, 0.88 g; 7.22 mmol), *N*-(3-dimethylaminopropyl)-*N*-ethylcarbodiimide hydrochloride (EDCI) (1.38 g; 7.22 mmol), and prop-2-en-1-ol (0.38 g; 6.62 mmol) were dissolved in *N*,*N*-dimethylformamide (100 mL). After the solution was stirred at room temperature for 12 h, the solvent was removed by rotary evaporation, and the residue was purified by column chromatography to give MDP (0.42 g, 28.3%) as a faint yellow oil. ^1^H NMR (CDCl_3_): *δ* 7.74–7.77 (m, 2H), 7.55–7.57 (m, 2H), 5.98–6.03 (m, 2H), 5.28–5.43 (m, 4H), 4.80–4.83 (m, 4H). ^13^C NMR (CDCl_3_): *δ* 167.21, 132.02, 131.83, 131.17, 128.98, 118.66, 66.29.

*Di(but-3-en-1-yl) (1,1’-biphenyl)-4,4’-dicarboxylate (MBD)*: 1,1’-Biphenyl-4,4’-dicarboxylic acid (0.50 g; 2.06 mmol), DMAP (0.60 g; 4.94 mmol), EDCI (0.95 g; 4.90 mmol), and but-3-en-1-ol (0.33 g; 4.54 mmol) were dissolved in *N*,*N*-dimethylformamide (100 mL). After the solution was stirred at room temperature for 12 h, the solvent was removed by rotary evaporation, and the residue was purified by column chromatography to give MBD (0.50 g, 34.5%) as a white solid. ^1^H NMR (CDCl_3_): *δ* 8.11 (d, 4H), 7.67 (d, 4H), 5.93–5.83 (m, 2H), 5.20–5.13 (m, 4H), 4.93 (t, 4H), 2.56–2.51 (m, 4H) ^13^C NMR (CDCl_3_): *δ* 166.32, 144.44, 134.10, 130.28, 129.96, 127.32, 117.57, 64.19, 33.28.

*Di(but-3-en-1-yl) naphthalene-2,6-dicarboxylate (MND)*: Naphthalene-2,6-dicarboxylic acid (0.50 g; 2.31 mmol), DMAP (0.67 g; 5.54 mmol), EDCI (1.06 g; 5.54 mmol), and but-3-en-1-ol (0.37 g; 5.09 mmol) were dissolved in *N*,*N*-dimethylformamide (100 mL). After the solution was stirred at room temperature for 12 h, the solvent was removed by rotary evaporation, and the residue was purified by column chromatography to give MND (0.37 g, 25.9%) as a white solid. ^1^H NMR (CDCl_3_): *δ* 8.67 (s, 2H), 8.18 (t, 2H), 8.07 (t, 2H), 5.91–6.02 (m, 2H), 5.22–5.30 (m, 4H), *δ* 4.51 (t, 4H), 2.61–2.68 (m, 4H). ^13^C NMR (CDCl_3_): *δ* 166.39, 134.66, 134.04, 130.68, 129.76, 129.66, 126.08, 117.59, 64.45, 33.29.

*PDA*: The polymer was synthesized by radial copolymerization^41^. MDP (0.02 g, 0.081 mmol), acrylic acid (5.86 g, 81.2 mmol), and AIBN (0.006 g) were dissolved in dry toluene (75 mL) under nitrogen atmosphere. After this solution was stirred at 70 °C for 17 h, the mixture was cooled to room temperature and the white solids were obtained by filtration. Then the crude product was washed with dichloromethane three times, which was dissolved in deionized water and dialyzed by dialysis tube (molecular weight cut-off = 2000) for 72 h. The solution was kept at 100 °C for 6 h, and the transparent polymer film was obtained.

*PBA*: Following the similar synthetic procedure for PDA, the reaction of MBD (0.02 g; 0.083 mmol), acrylic acid (5.95 g, 82.57 mmol), and AIBN (0.006 g) in dry toluene solution (75 mL) for 17 h yielded PBA as a transparent film.

*PNA*: Following the similar synthetic procedure for PDA, the reaction of MND (0.02 g; 0.062 mmol), acrylic acid (4.45 g, 61.67 mmol), and AIBN (0.006 g) in dry toluene solution (75 mL) for 17 h yielded PNA as a transparent film.

*PDNA*: Following the similar synthetic procedure for PDA, the reaction of MND (0.0013 g; 0.0041 mmol), MDP (0.2 g; 0.82 mmol), acrylic acid (2.95 g, 41.0 mmol), and AIBN (0.008 g) in dry toluene solution (75 mL) for 17 h yielded PDNA as a transparent film.

*PDBA*: Following the similar synthetic procedure for PDA, the reaction of MDP (0.045 g; 0.185 mmol), MBD (0.013 g; 0.037 mmol), acrylic acid (2.60 g, 37.0 mmol), and AIBN (0.008 g) in dry toluene solution (75 mL) for 17 h yielded PDBA as a transparent film.

## Supplementary information

Supplementary Information

Description of Additional Supplementary Files

Supplementary Movie 1

Supplementary Movie 2

## Data Availability

The authors declare that the data supporting the findings of this study are available within the article and its Supplementary Information. Extra data are available from the corresponding authors upon reasonable request.
