# Supplementary material for: Color-tunable ultralong organic room temperature phosphorescence from a multicomponent copolymer
Source: Nat Commun. 2020 Feb 18;11:944. doi: 10.1038/s41467-020-14792-1 (PMC7029031; doi:10.1038/s41467-020-14792-1)
Supplement: Supplementary file 1 — Supplementary Information [file 41467_2020_14792_MOESM1_ESM.pdf]

Supplementary Information

Color-Tunable Ultralong Organic Room Temperature Phosphorescence from a Multicomponent Copolymer

Gu et al.

Supplementary Methods

Reagents and Materials

Unless otherwise stated, all reagents used in the experiments were purchased from commercial sources without further purification. Toluene was dried by sodium through distillation, with benzophenone as a chromogenic reagent. For flash column chromatography, silica gel with 200 ~ 300 mesh was used.

Measurements

Nuclear magnetic resonance (^{1}H NMR and ^{13}C NMR) spectra were obtained on a Bruker Ultra Shield Plus 300 MHz spectrometer. Chemical shift was relative to tetramethylsilane (TMS) as the internal standard. Resonance patterns were reported with the notation s (singlet), d (double), t (triplet), q (quartet), and m (multiplet). Ultraviolet absorption spectra were obtained by SHIMADZU UV-1750. Steady-state fluorescence/phosphorescence spectra and excitation spectra were measured using HitachiF-7100, and all phosphorescence spectra were record with a delay of 5 ms. The lifetime and time-resolved emission spectra were obtained on Edinburgh FLSP 920 fluorescence spectrophotometer equipped with a xenon arc lamp (Xe 900), a nanosecond hydrogen flash-lamp (nF 920), or a microsecond flash-lamp (μF900). Photoluminescence efficiency was collected on a Hamamatsu Absolute PL Quantum Yield Spectrometer C11347. Wide-angle X-ray scattering patterns were achieved from a Xenocs Nanoinxider with Cu-Ka microsource (40 mm) at 30W. Powder X-ray diffraction patterns were recorded on a Rigaku D/max 2250VB/PC diffraction with Cu-Kα radiation (λ = 1.5406 Å). Aqueous gel permeation chromatography was performed on Viscotek TDA305 max. 0.1 mol/L $NaNO_3$ solution was used as the mobile phase at 0.7 mL min^{-1} flow rate. PEO was used as the calibration standard. Luminescent photos and videos were taken by a Cannon EOS 700D single lens digital camera.

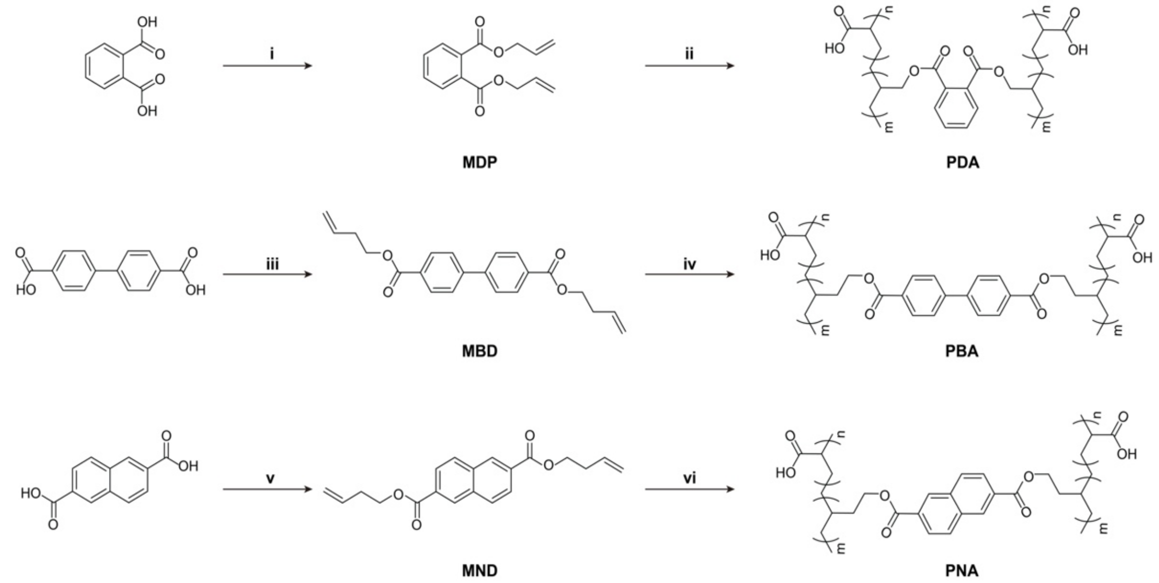

Supplementary Figure 1. Synthetic routes of binary copolymers. (i) EDCI, DMAP, Prop-2-en-1-ol, room temperature, 12 h; (iii) and (v) EDCI, DMAP, But-3-en-1-ol, room temperature, 12 h;[1,2] (ii), (iv) and (vi) Acrylic acid. AIBN, toluene, 70 °C, 17h.

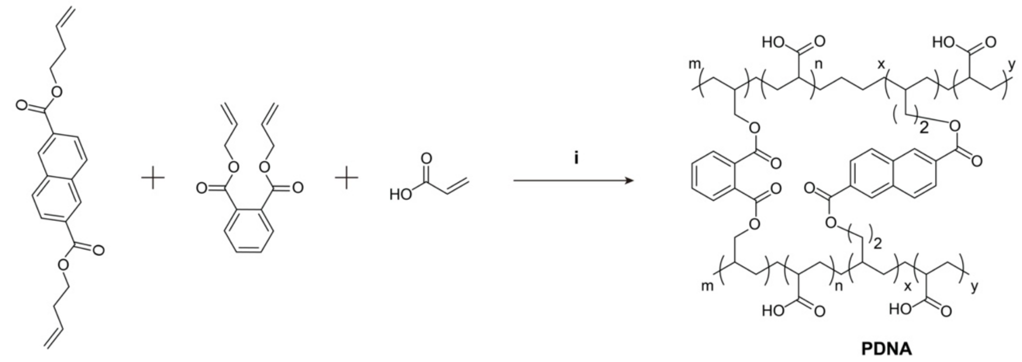

Supplementary Figure 2. Synthetic route of multicomponent copolymer PDNA. (i) AIBN, toluene, 70 °C, 17h.

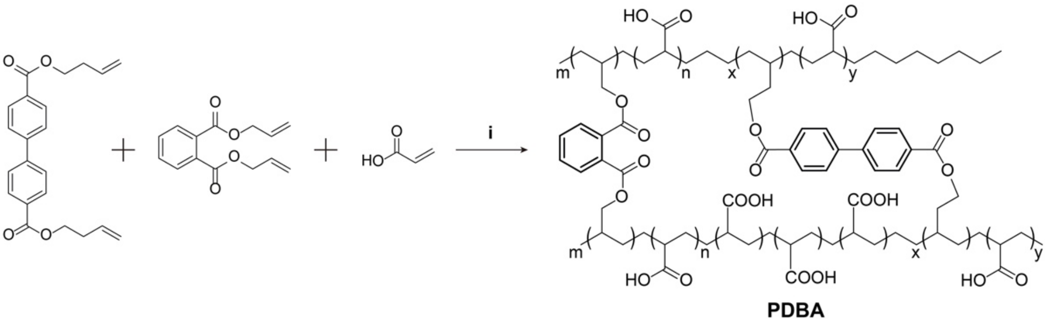

Supplementary Figure 3. Synthetic route of multicomponent copolymer PDBA. (i) AIBN, toluene, 70 °C, 17h.

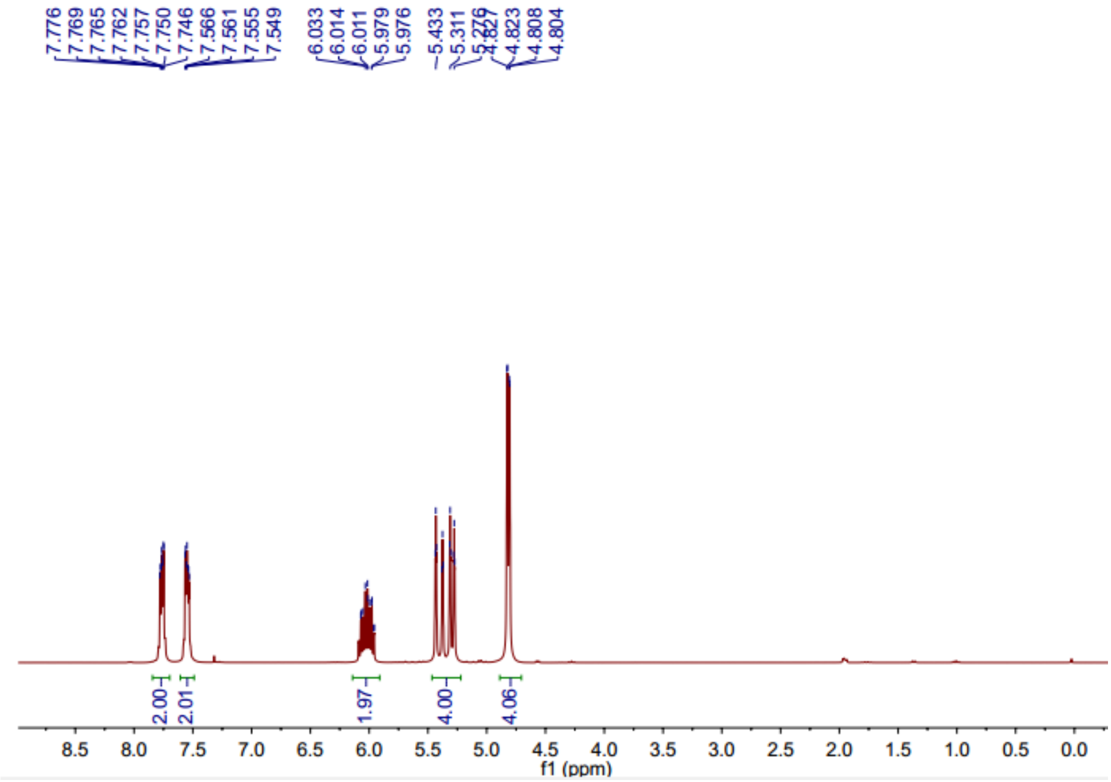

Supplementary Figure 4. ^{1}H NMR spectrum of MDP molecule in $CDCl_3$.

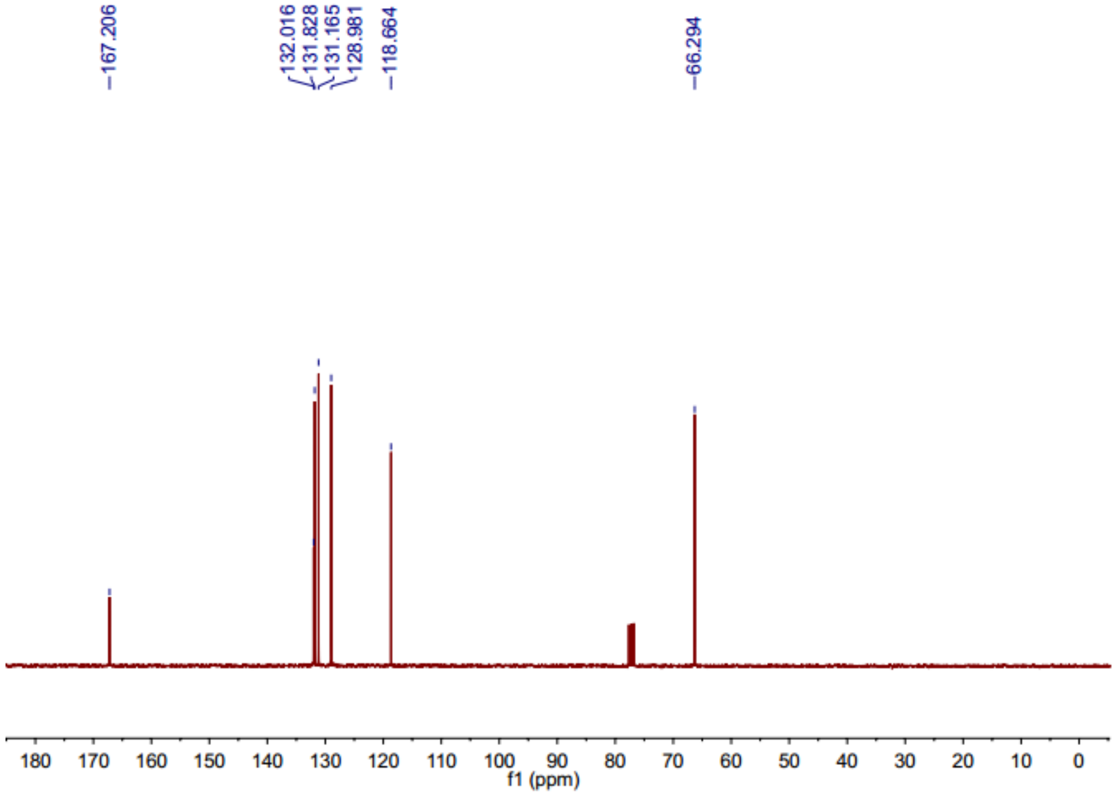

Supplementary Figure 5. ^{13}C NMR spectrum of MDP molecule in $CDCl_3$.

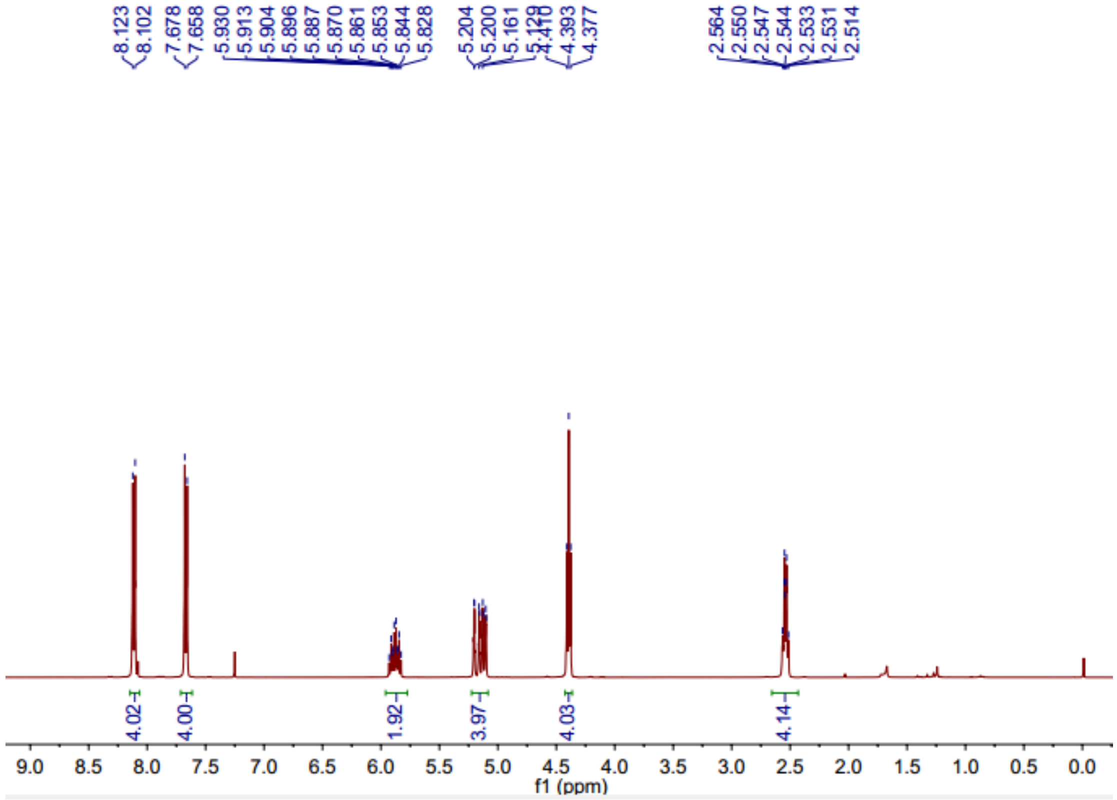

Supplementary Figure 6. ^{1}H NMR spectrum of MBD molecule in $CDCl_3$.

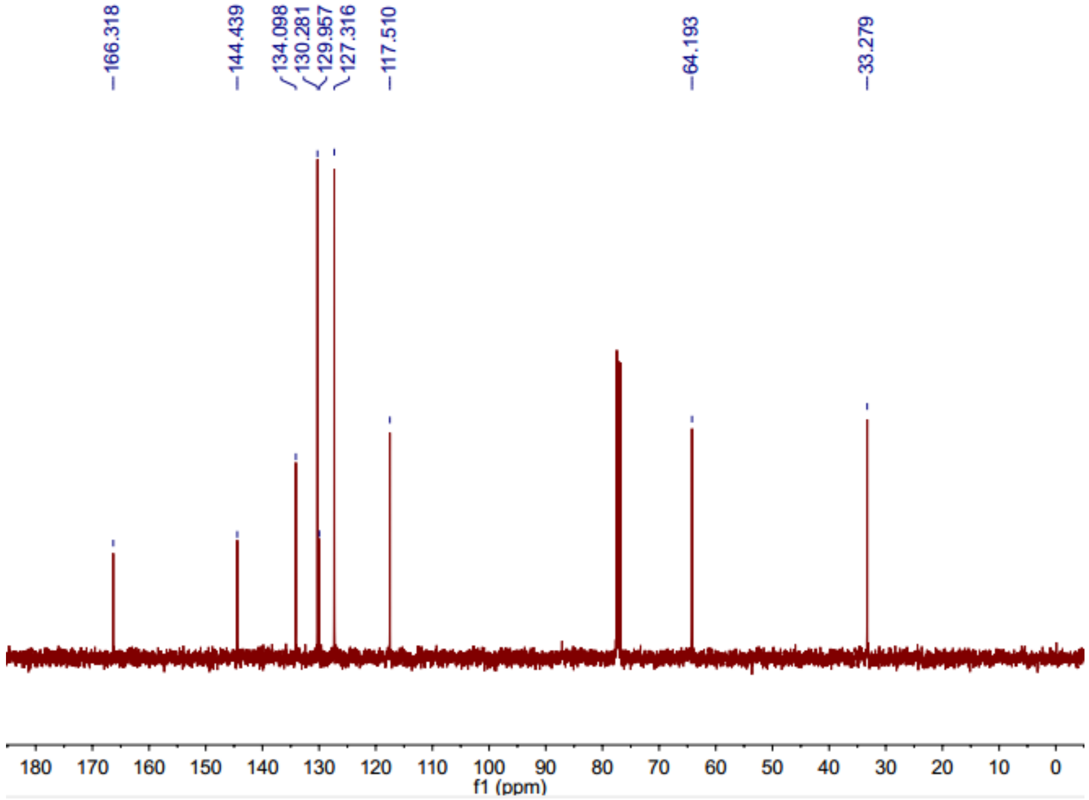

Supplementary Figure 7. ^{13}C NMR spectrum of MBD molecule in $CDCl_3$.

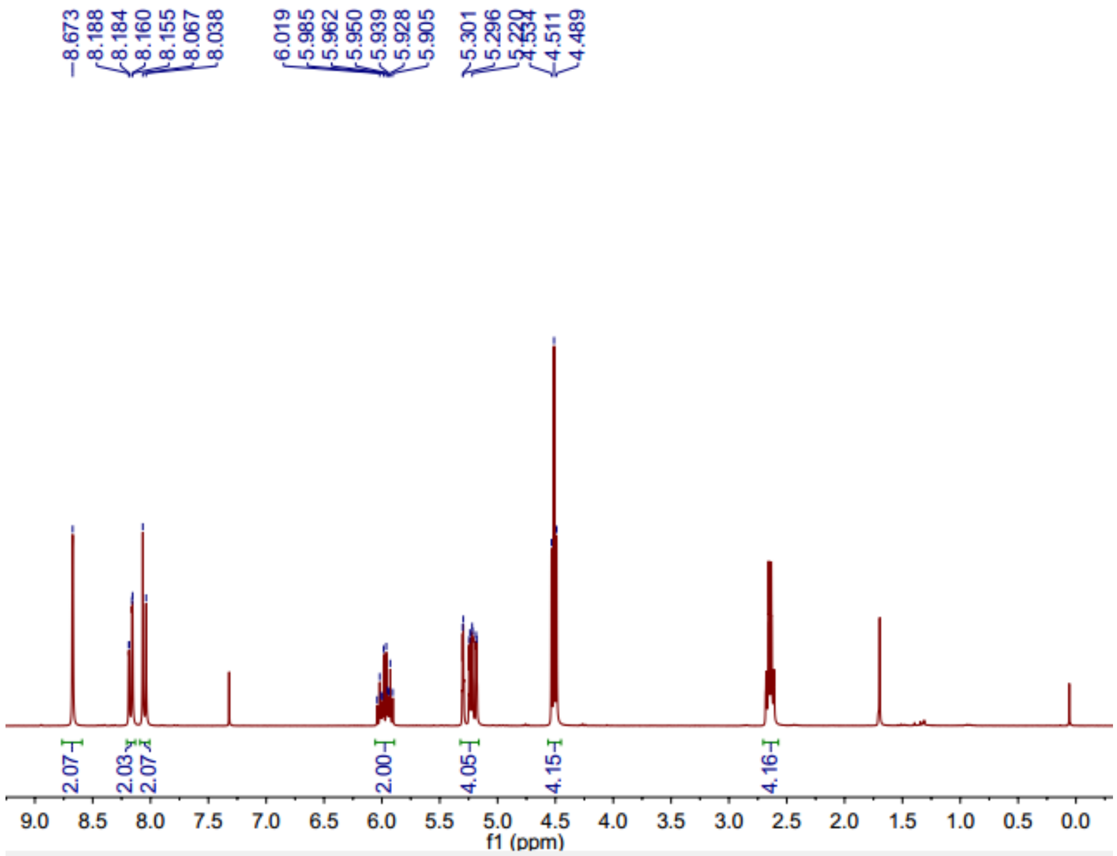

Supplementary Figure 8. ^{1}H NMR spectrum of MND molecule in $CDCl_3$.

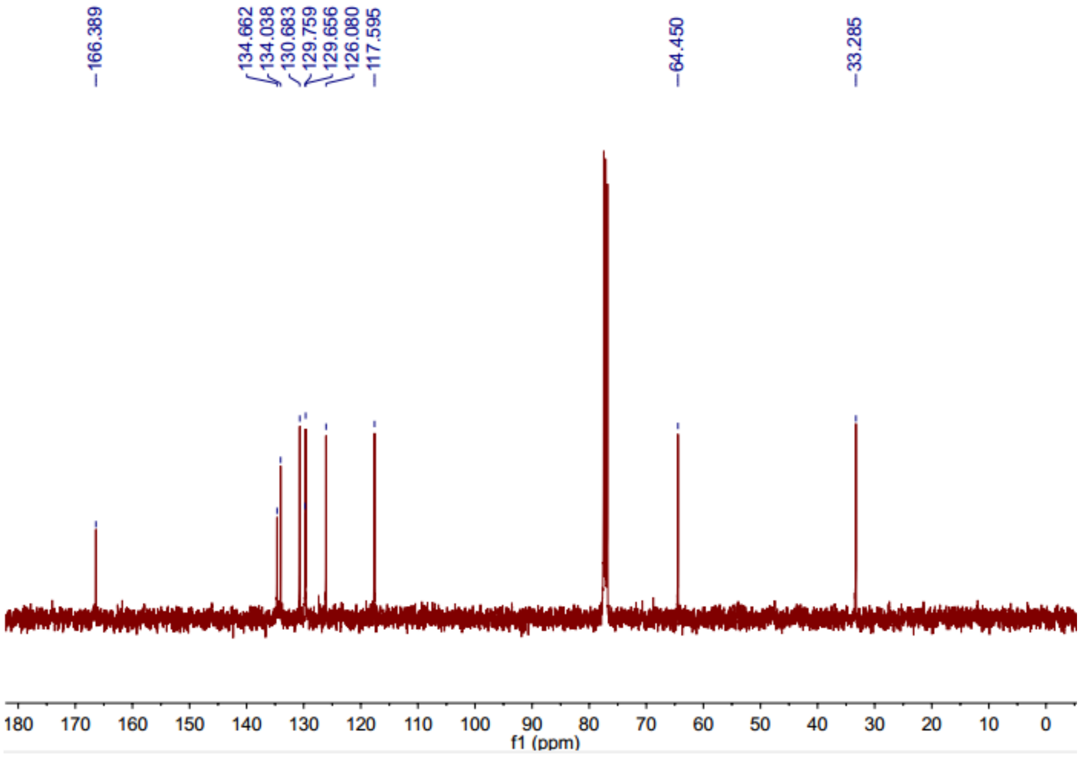

Supplementary Figure 9. ^{13}C NMR spectrum of MND molecule in $CDCl_3$.

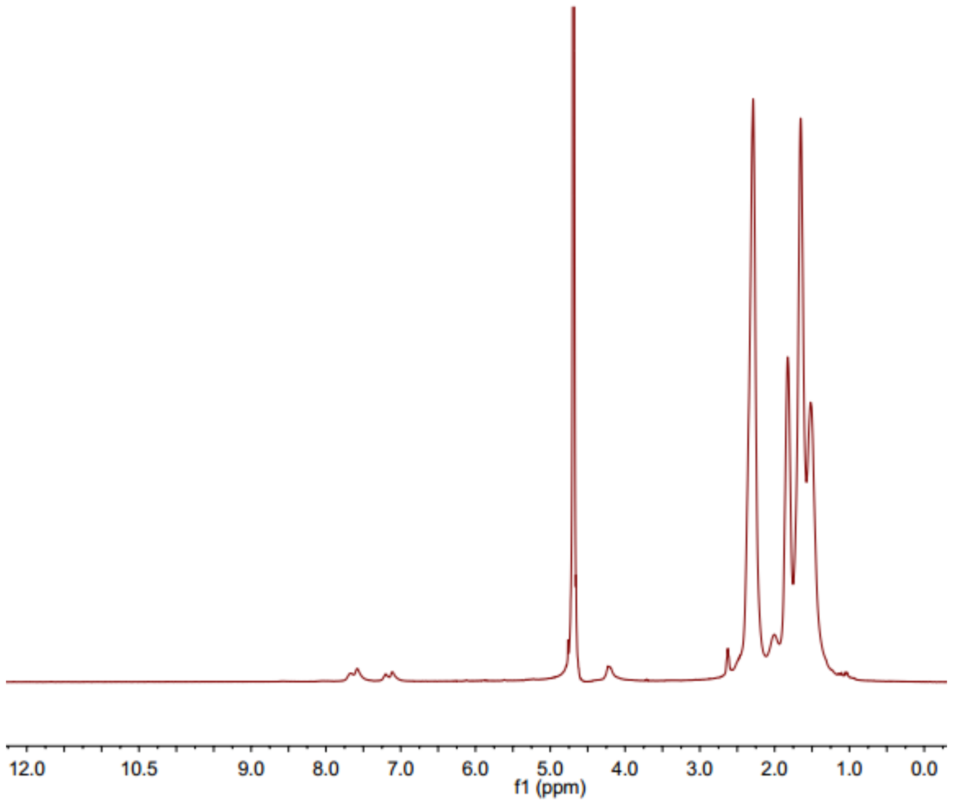

Supplementary Figure 10. ^1H NMR spectrum of polymer PDNA in D_2O.

Supplementary Table 1. Characterizations of polymers.[a]

Sample	PDNA	PDNA-5	PDNA-10	PDNA-20	PDNA-40	PDNA-100	PDBA
**Mw (Da)**	1,059,000	358,598	184,866	1,349,000	78,420	20,314	145,229
**Mn (Da)**	67,295	71,197	40,039	562,752	30,236	11,692	43,441
**PDI**	15.70	5.04	4.62	2.40	2.60	1.74	3.34

[a]Mw and Mn were determined by aqueous GPC.

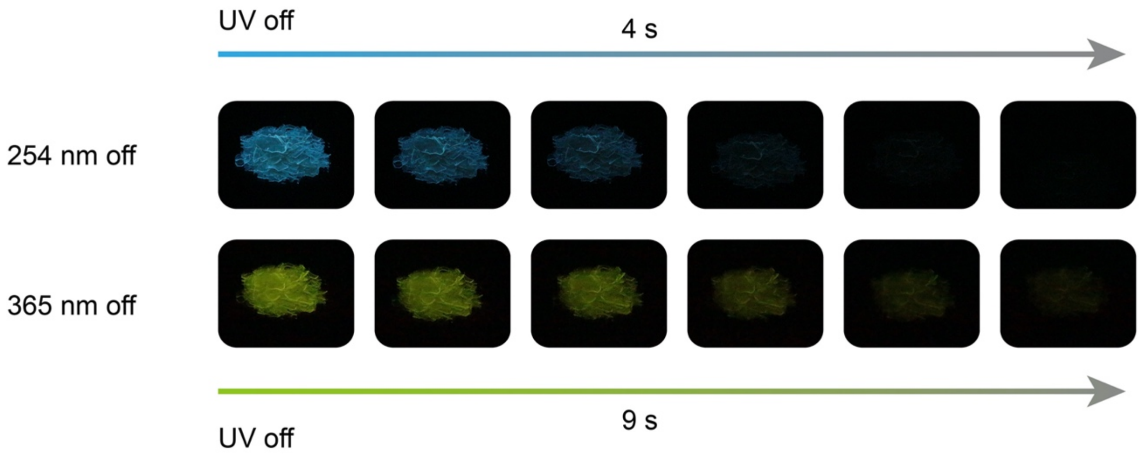

Supplementary Figure 11. Photographs of PDNA polymer film monitoring the deactivation process of UOP excited at 254 and 365 nm, respectively.

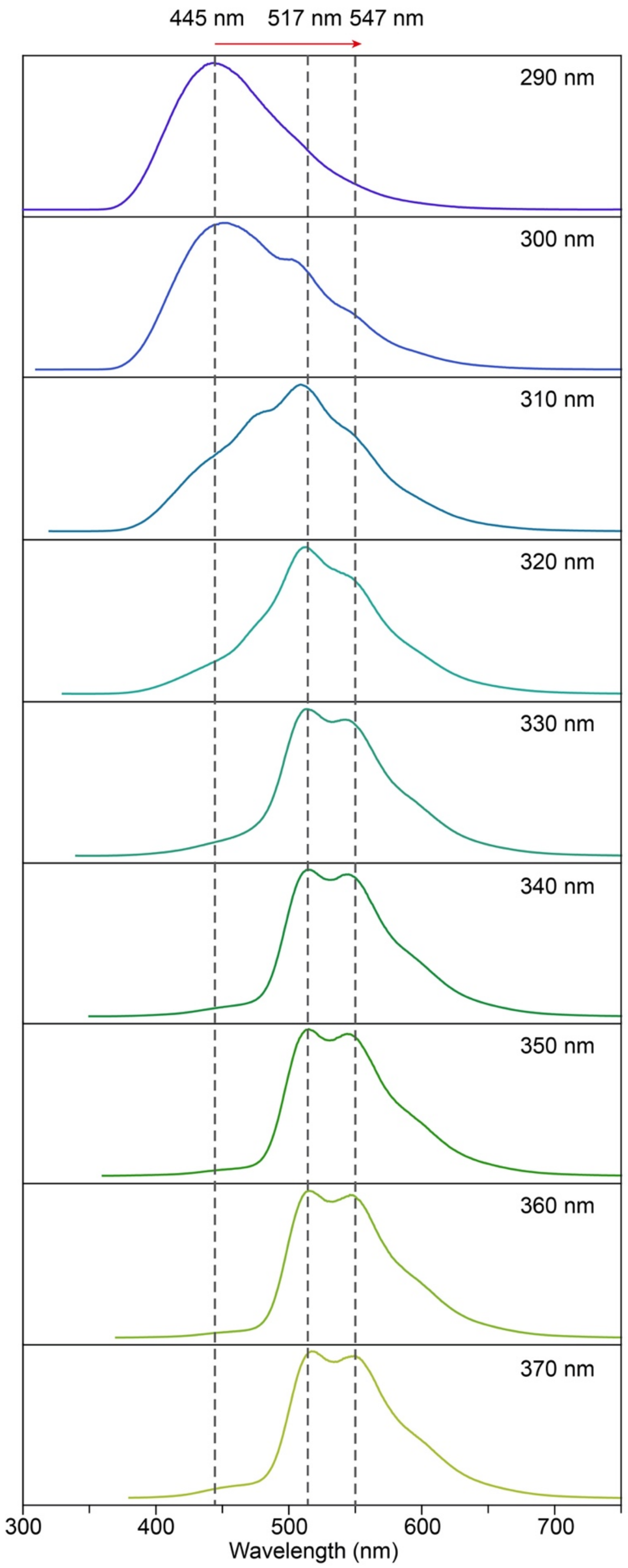

Supplementary Figure 12. Normalized phosphorescence spectra of PDNA polymer film upon excitation at 290, 300, 310, 320, 330, 340, 350, 360 and 370 nm under ambient conditions, respectively.

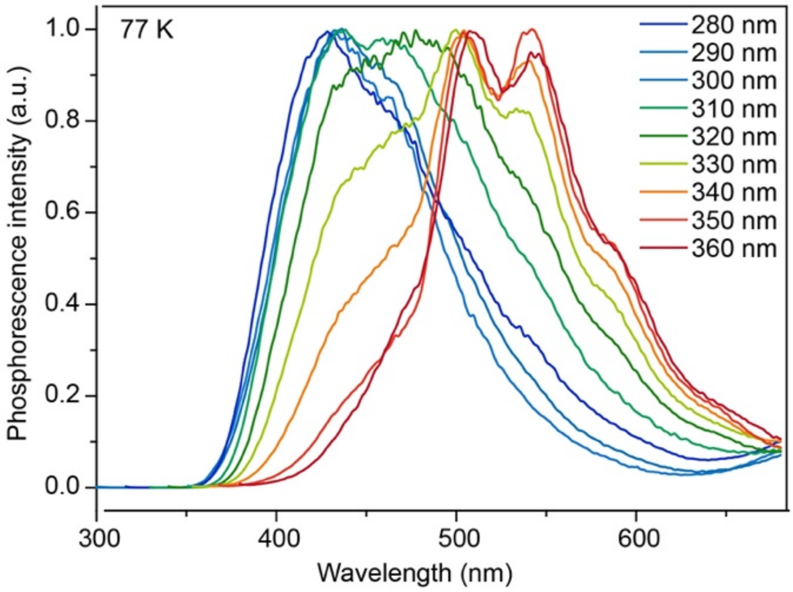

Supplementary Figure 13. Phosphorescence emission spectra of PDNA polymer film at 77 K excited by 280, 290, 300, 310, 320, 330, 340, 350 and 360 nm, respectively.

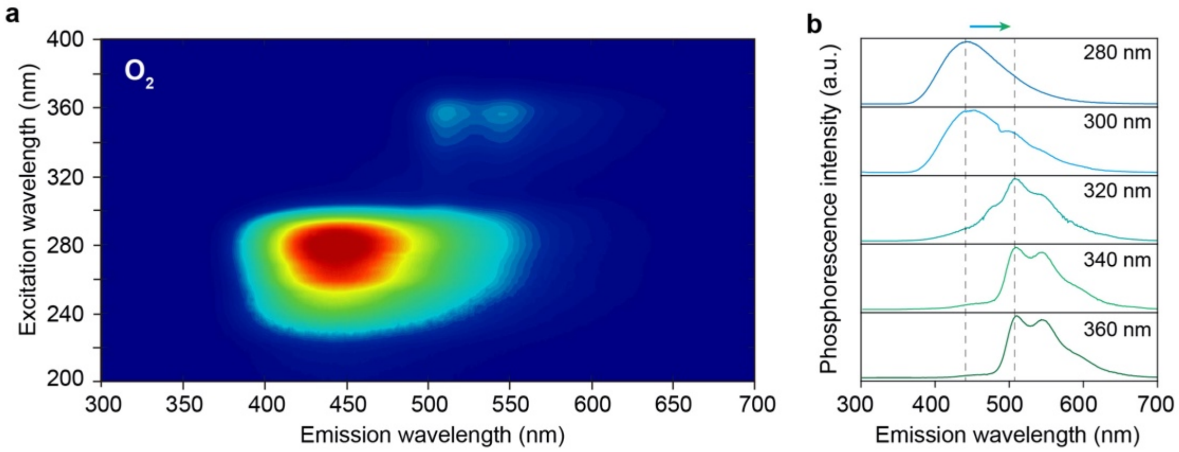

Supplementary Figure 14. **a**, Phosphorescence-excitation mapping of polymer PDNA under oxygen atmosphere. The color change from red to blue indicates the decrease in emission intensity. **b**, UOP spectral profiles of polymer PDNA measured under different excitation wavelengths.

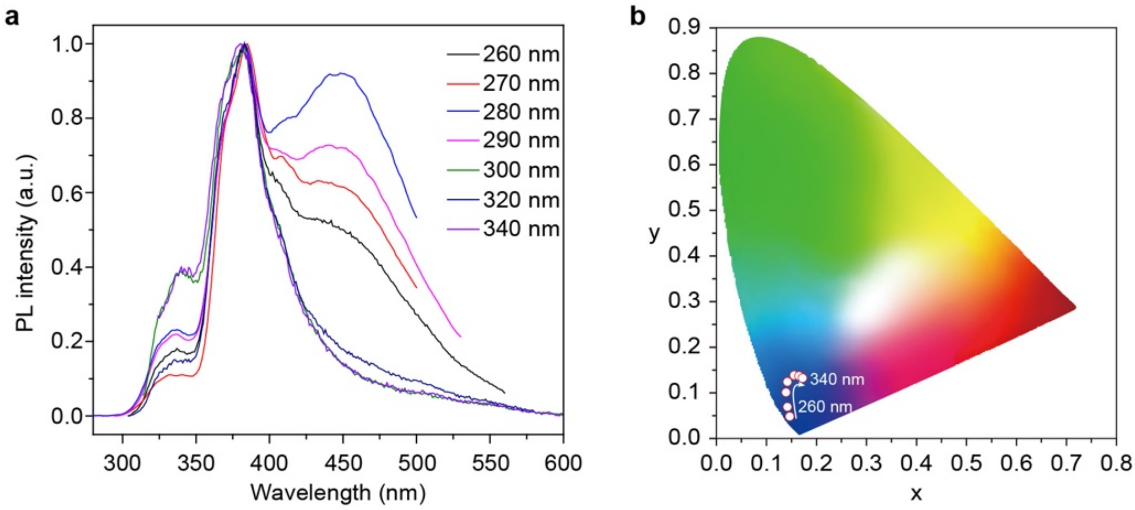

Supplementary Figure 15. **a**, Normalized photoluminescence (PL) spectra of PDNA polymer film upon excitation at 260, 270, 280, 290, 300, 320 and 340 nm under ambient conditions. **b**, CIE chromaticity coordinates of the photoluminescence for polymer PDNA at various excitation wavelengths.

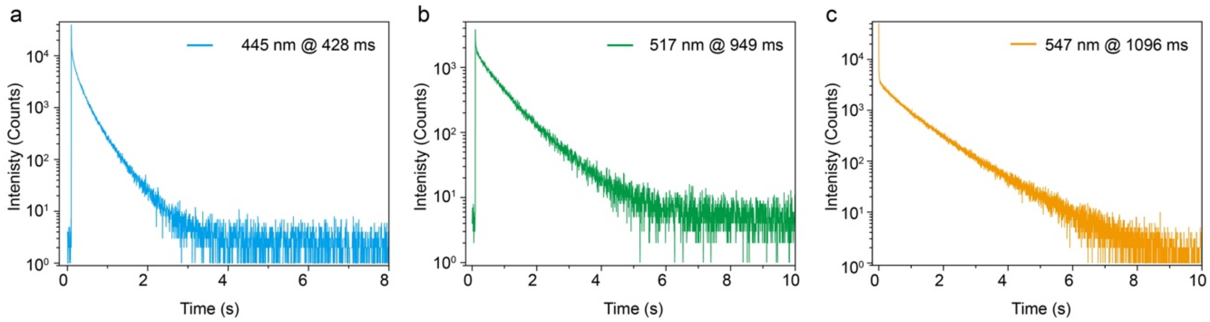

Supplementary Figure 16. **a-c**, Lifetime decay profiles of UOP bands at 445, 517 and 547 nm for PDNA polymer film upon excitation at 280 and 360 nm under room temperature, respectively.

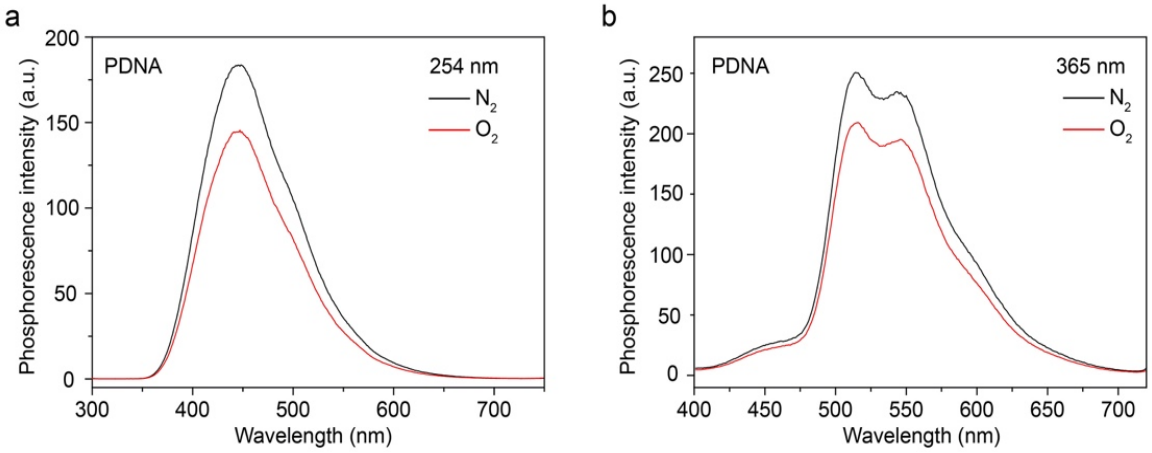

Supplementary Figure 17. **a**,**b**, Phosphorescence spectra of PDNA polymer film excited by 254 and 365 nm under nitrogen (black line) and oxygen (red line) atmosphere at room temperature, respectively.

Supplementary Table 2. Photoluminescence and phosphorescence efficiencies of the polymer films PDNA, PDNA-5, PDNA-10, PDNA-20, PDNA-40 and PDNA-100 under ambient conditions.

Compounds	$\varphi_{pl.}$ (%)	$\varphi_{Phos.}$ (%)
PDNA	39.8	23.2
PDNA-5	22.4	13.0
PDNA-10	64.7	37.5
PDNA-20	65.7	37.5
PDNA-40	45.5	26.4
PDNA-100	50.3	29.2

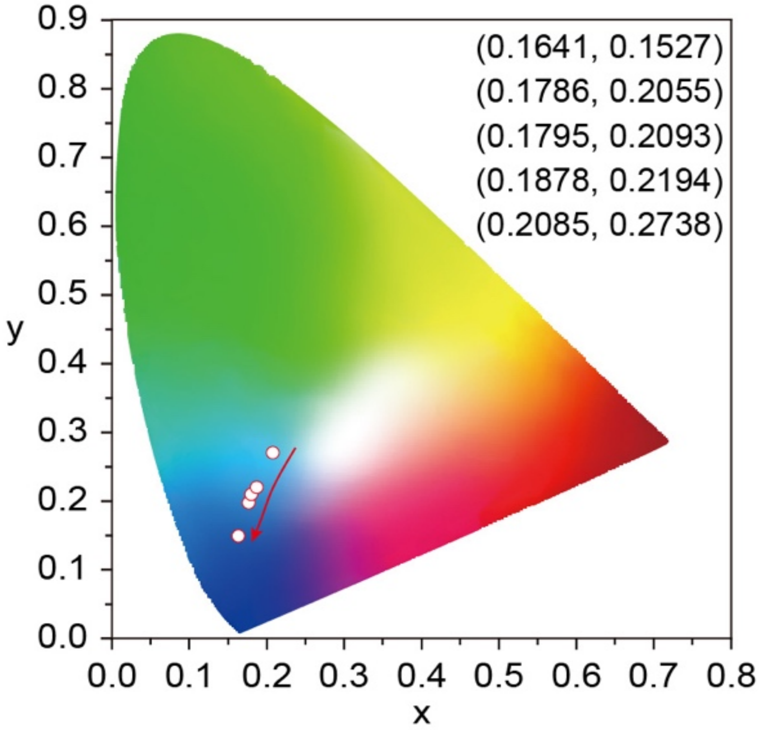

Supplementary Figure 18. Trajectory of color modulation, recorded by the change in the molar feed ratio of MND : MDP : acrylic acid of 1:5:1000 (PDNA-5), 1:10:1000 (PDNA-10), 1:20:1000 (PDNA-20), 1:40:1000 (PDNA-40) and 1:100:1000 (PDNA-100) excited at 254 nm, in the CIE coordinate diagram.

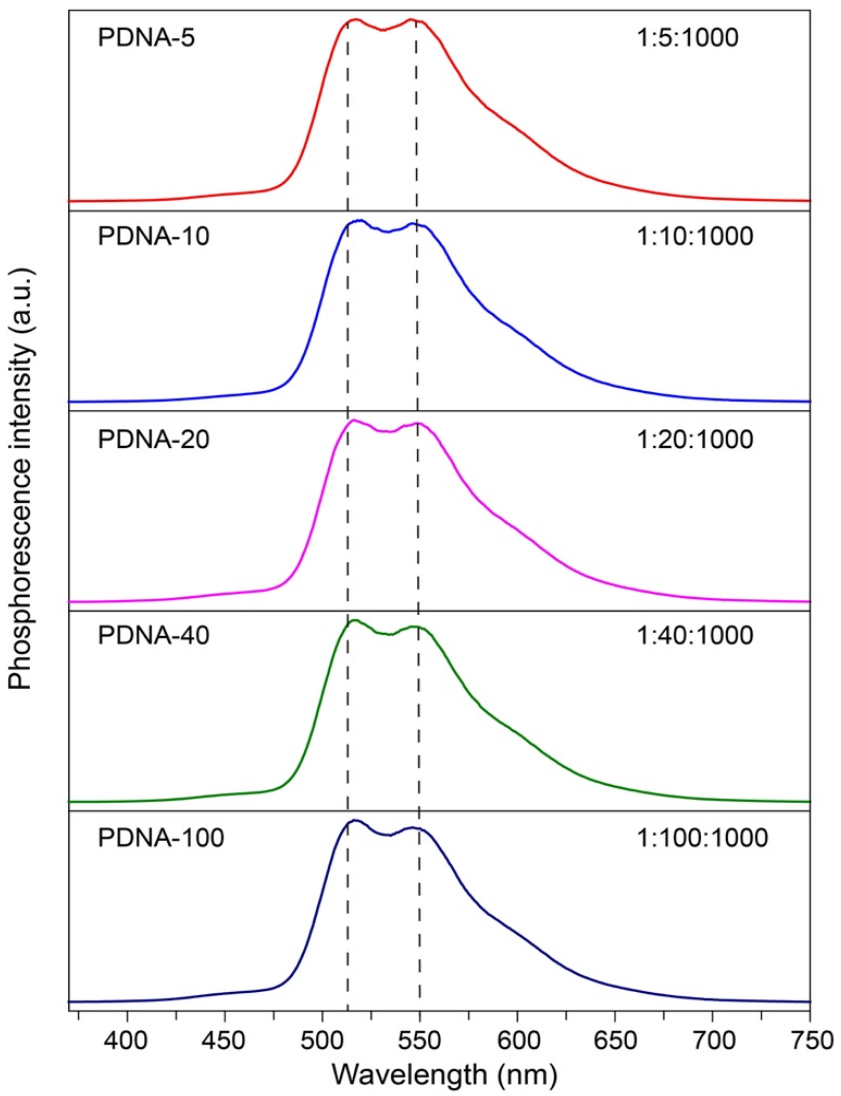

Supplementary Figure 19. Phosphorescence spectra of PDNA polymer film with different molar feed ratio of MND : MDP : acrylic acid from 1:5:1000 to 1:100:1000 excited at 365 nm at room temperature, respectively.

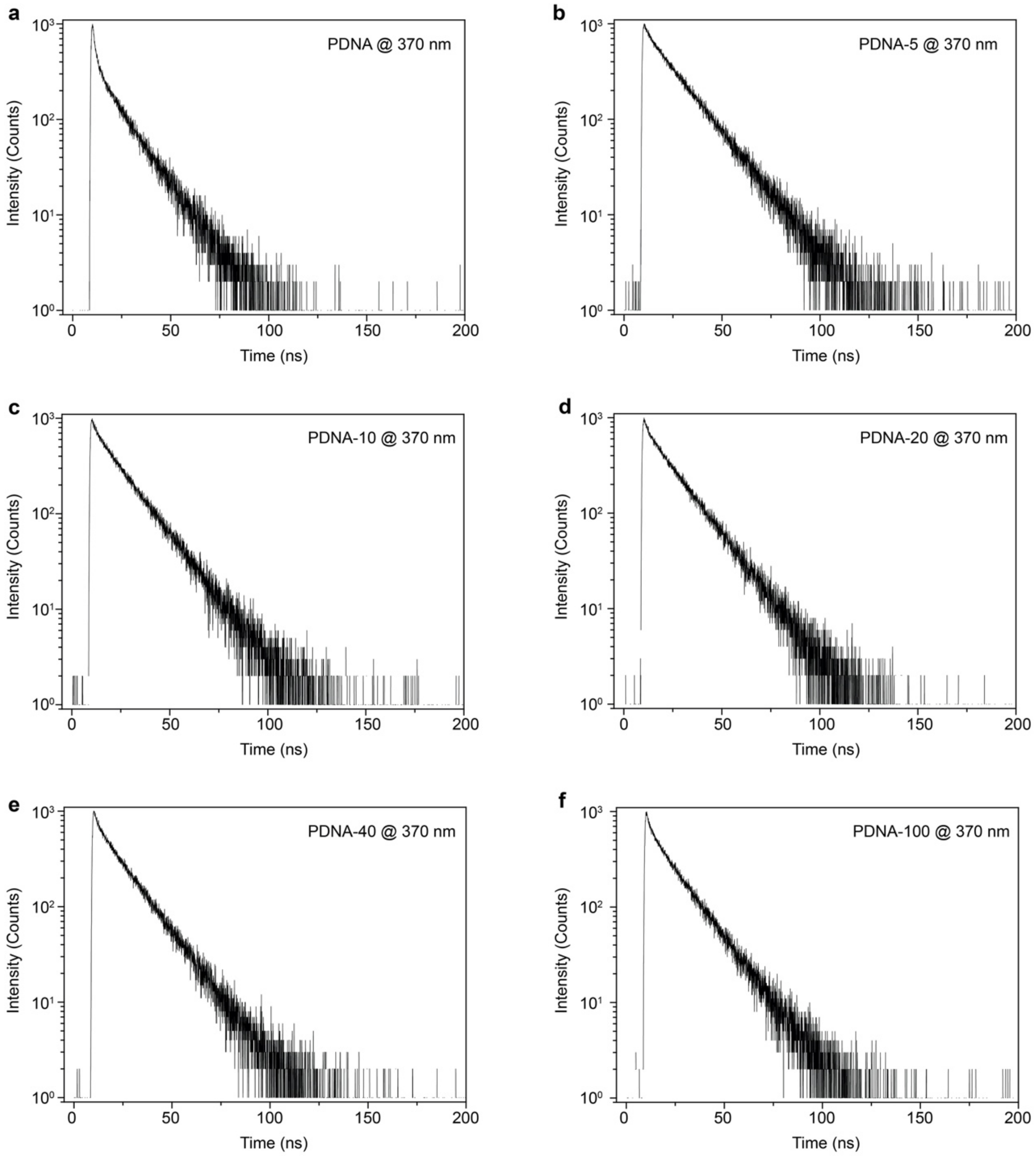

Supplementary Figure 20. **a-f**, Lifetime decay profiles of UOP band at 370 nm for PDNA polymer film with different molar feed ratio of MND : MDP : acrylic acid of 1:200:10000 (PDNA), 1:5:1000 (PDNA-5), 1:10:1000 (PDNA-10), 1:20:1000 (PDNA-20), 1:40:1000 (PDNA-40) and 1:100:1000 (PDNA-100) upon excitation at 300 nm under room temperature, respectively.

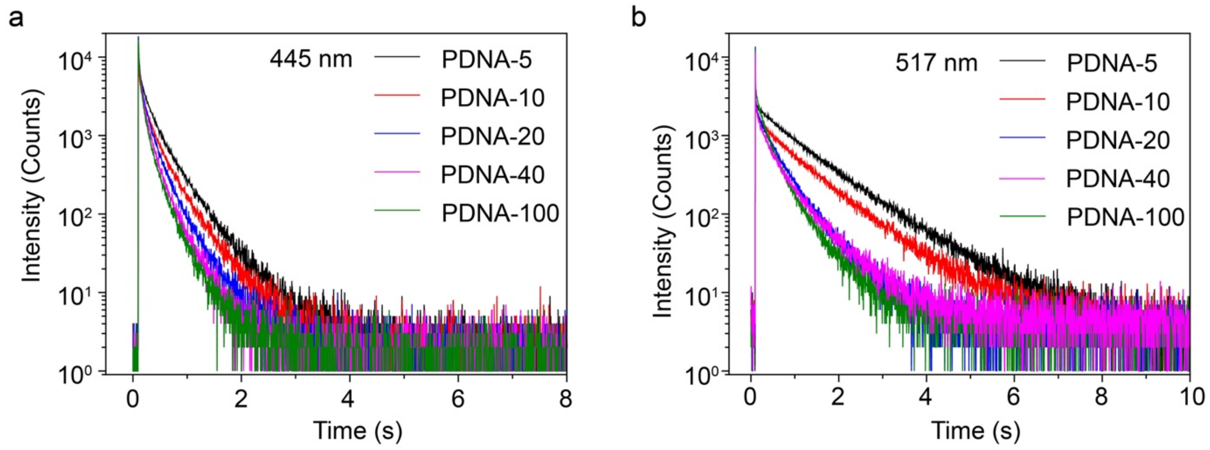

Supplementary Figure 21. **a**,**b**, Lifetime decay profiles of UOP bands at 445 and 517 nm for PDNA polymer film with different molar feed ratio of MND : MDP : acrylic acid from 1:5:1000 to 1:100:1000 upon excitation at 280 and 360 nm under room temperature, respectively.

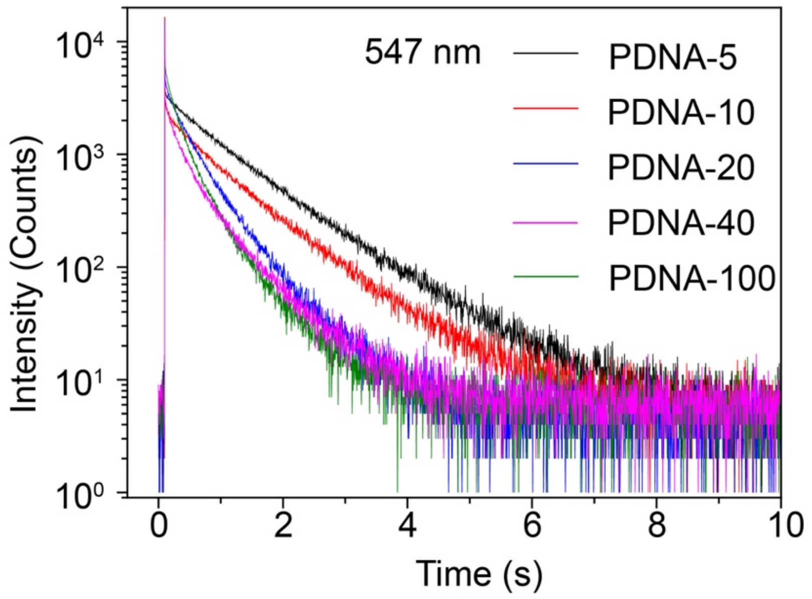

Supplementary Figure 22. Lifetime decay profiles of UOP band at 547 nm for PDNA polymer film with different molar feed ratio of MND : MDP : acrylic acid from 1:5:1000 to 1:100:1000 upon excitation at 360 nm under room temperature, respectively.

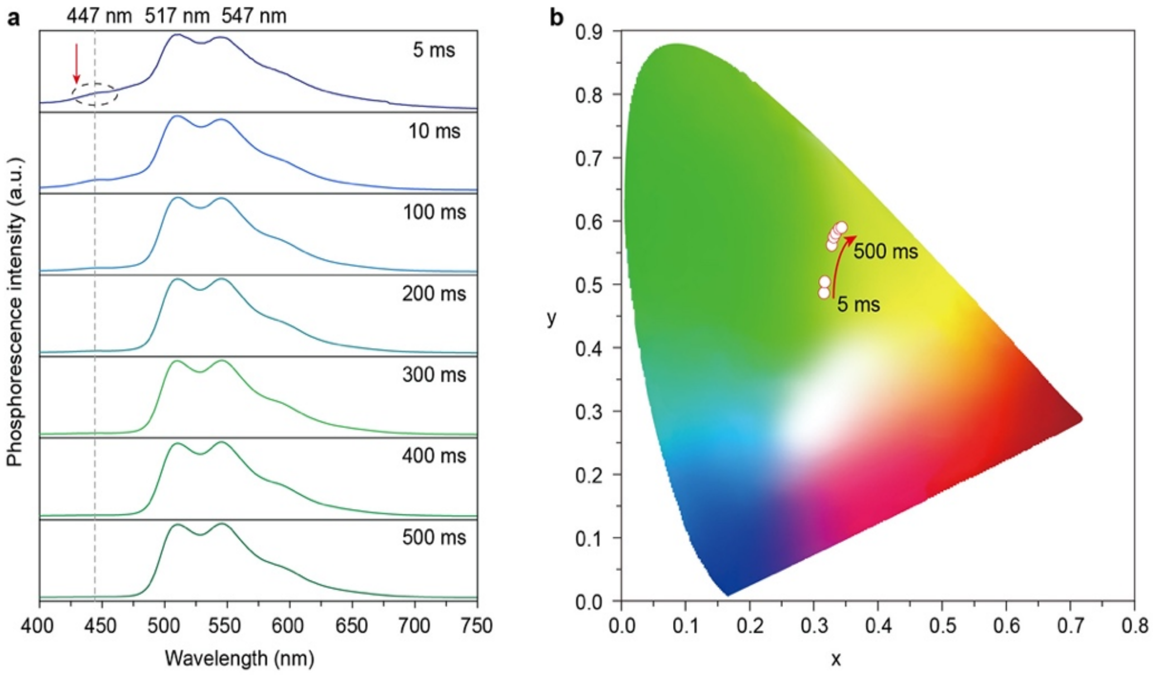

Supplementary Figure 23. **a**, Phosphorescence spectra of PDNA at delay times of 5, 10, 100, 200, 300, 400 and 500 ms, respectively. **b**, CIE chromaticity coordinates of the ultralong luminescence for polymer PDNA at various delay times.

Supplementary Table 3. Photoluminescence lifetimes (τ) of polymer films under ambient conditions.[a]

Compound	Wavelength (nm)	Fluorescence				Phosphorescence					
		τ_1 (ns)	A_1 (%)	τ_2 (ns)	A_2 (%)	τ_1 (ms)	A_1 (%)	τ_2 (ms)	A_2 (%)	τ_3 (ms)	A_3 (%)
PDNA-5	370	2.34	2.36	16.46	97.64	-	-	-	-	-	-
	445	-	-	-	-	304.6	7.20	176.9	45.02	479.7	47.78
	517	-	-	-	-	182.0	0.62	567.8	24.12	1222	75.26
	547	-	-	-	-	186.9	0.43	612.8	27.03	1024	72.54
PDNA-10	370	15.06	100	-	-	-	-	-	-	-	-
	445	-	-	-	-	232.9	8.30	150.9	43.14	434.2	48.56
	517	-	-	-	-	261.3	2.03	463.5	21.17	1070	76.80
	547	-	-	-	-	269.4	11.64	1012	88.36		
PDNA-20	370	14.91	100	-	-	-	-	-	-	-	-
	445	-	-	-	-	216.1	15.83	119.3	49.05	353.2	35.12
	517	-	-	-	-	472.5	6.17	296.2	49.33	826.5	44.50
	547	-	-	-	-	580.2	5.98	292.9	47.51	804.9	46.51
PDNA-40	370	1.77	3.91	15.22	96.09	-	-	-	-	-	-
	445	-	-	-	-	189.3	18.69	100.2	51.06	312.3	30.25
	517	-	-	-	-	290.4	3.18	313.3	50.74	709.8	46.08
	547	-	-	-	-	245.3	38.72	650.1	61.28	-	-
PDNA-100	370	14.28	100	-	-	-	-	-	-	-	-
	445	-	-	-	-	143.7	19.02	843.1	49.51	282.0	31.47
	517	-	-	-	-	377.3	6.61	227.4	58.11	636.2	35.28
	547	-	-	-	-	537.3	7.60	243.3	60.00	677.1	32.40

[a]Determined from the fitting function of $I(t) = A_1e^{-t/\tau_1} + A_2e^{-t/\tau_2} + A_3e^{-t/\tau_3}$ according to the photoluminescence decay curves.

Supplementary Note 1

Additional photophysical properties of monomer and polymer in solution and film. The photophysical properties of compounds MDP, MND and MBD were fully investigated through absorption and photoluminescence spectra in solution. In dilute chloroform solution (1 x 10^{-5} M), both monomer MDP and MND show two absorption bands at 241 and 276 nm, as well as 243 and 285 nm ascribing to the π-π* and n-π* transition of benzene and naphthalene units, respectively. For MBD, a broad absorption band at 285 nm was observed. As shown in Supplementary Figure 24, the compound MDP displays a structureless band at 334 nm. For MND, there are two emission bands with peaks at 361 and 377 nm. For compound MBD, a broad structureless emission band with a peak at 335 nm and a shoulder at around 322 nm were observed.

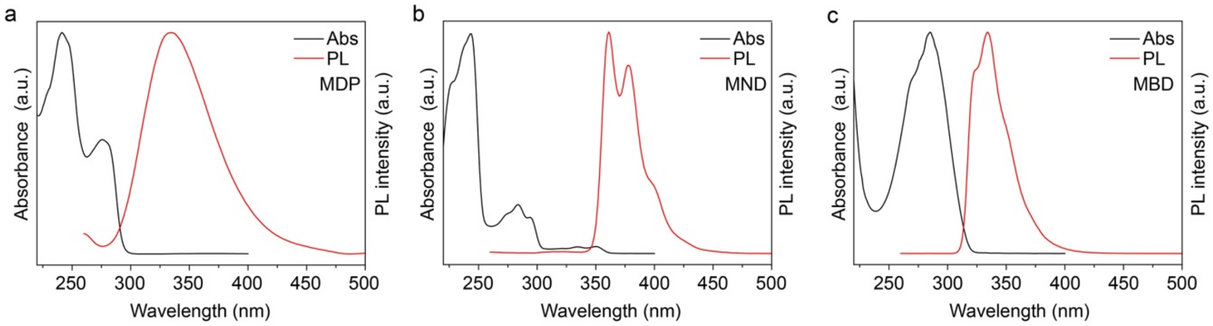

Supplementary Figure 24. **a-c**, Normalized absorption (black line) and steady-state photoluminescent (red line) spectra of MDP, MND and MBD in chloroform solution (1 x 10^{-5} M) at room temperature, respectively.

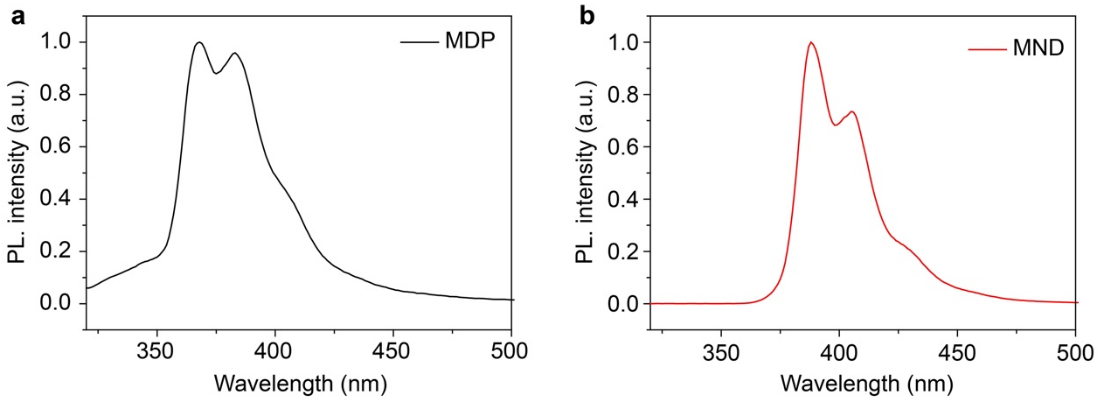

Supplementary Figure 25. **a**,**b**, Normalized photoluminescent (PL) spectra of monomers MDP and MND upon excitation at 300 nm in the solid state at room temperature.

Supplementary Note 2

Effect of molar feed ratio on ultralong organic room temperature phosphorescence. The effect of molar feed ration on the phosphorescence properties of PDA and PNA was explored, with the molar ratio of MDA:AA and MNA:AA increased from 1:50, 1:100, 1:500 to 1:1000. These polymers show similar blue and yellow phosphorescence emission capable of a long lifetime arranging from 442 to 520.6 ms as well as 524 to 1000 ms, when excited by the excitation of 280 and 300 nm, respectively. This result suggests that the variation of molar feed ratio has no influence on the phosphorescence color in polymers PDA and PNA.

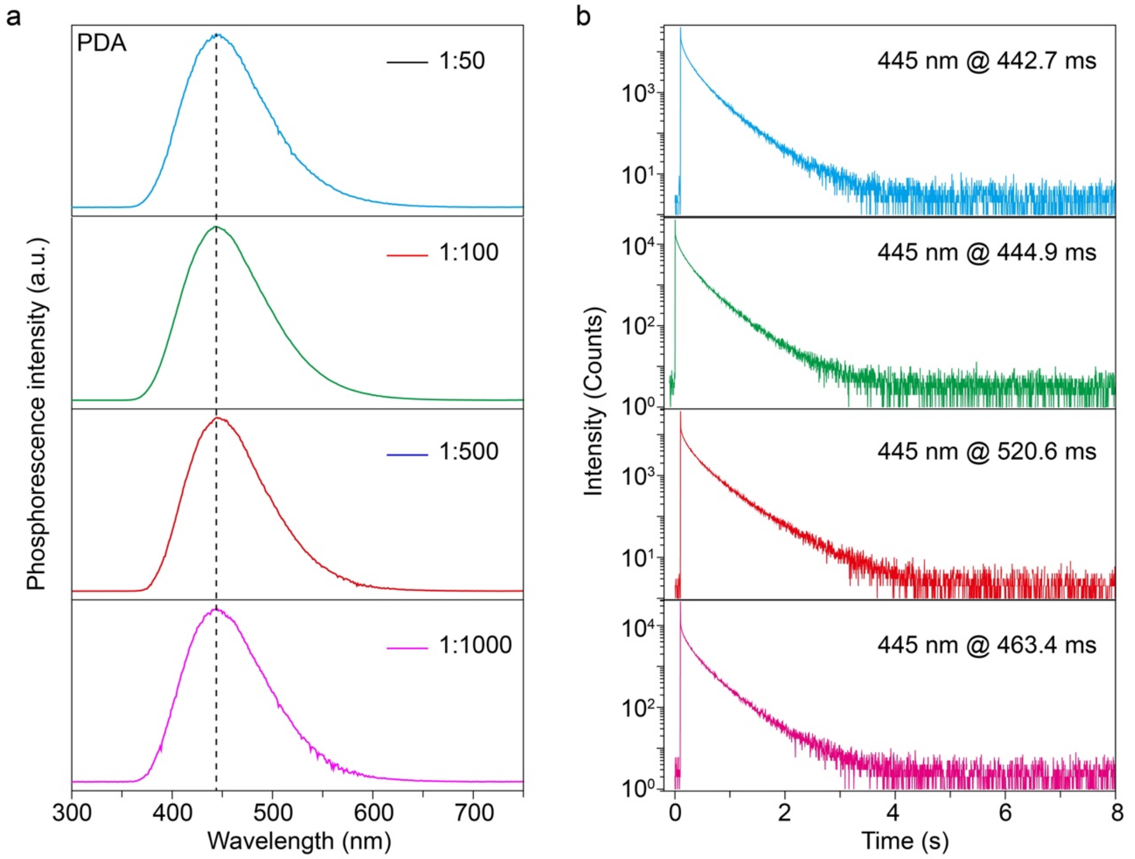

Supplementary Figure 26. **a**, Phosphorescence spectra of PDA polymer film with different molar feed ratio of MDP : acrylic acid from 1:50 to 1:1000 excited at 280 nm under room temperature. **b**, Lifetime decay profiles of UOP band at 445 nm for PDA polymer film with different molar feed ratio of MDP : acrylic acid from 1:50 to 1:1000 upon excitation at 280 nm under room temperature.

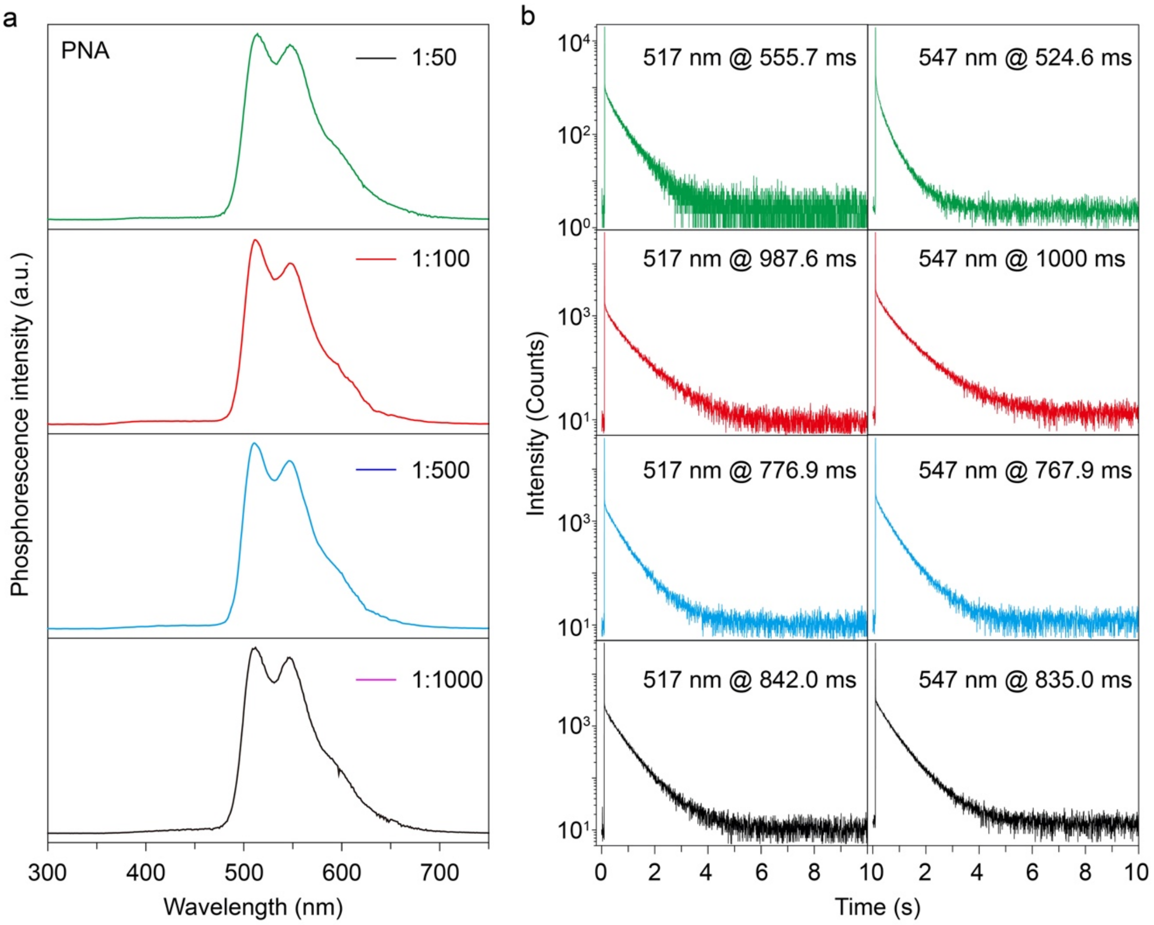

Supplementary Figure 27. **a**, Phosphorescence spectra of PNA polymer film with different molar feed ratio of MND : acrylic acid from 1:50 to 1:1000 excited at 300 nm under room temperature. **b**, Lifetime decay profiles of UOP bands at 517 and 547 nm for PNA polymer film with different molar feed ratio of MND : acrylic acid from 1:50 to 1:1000 upon excitation at 300 nm under room temperature.

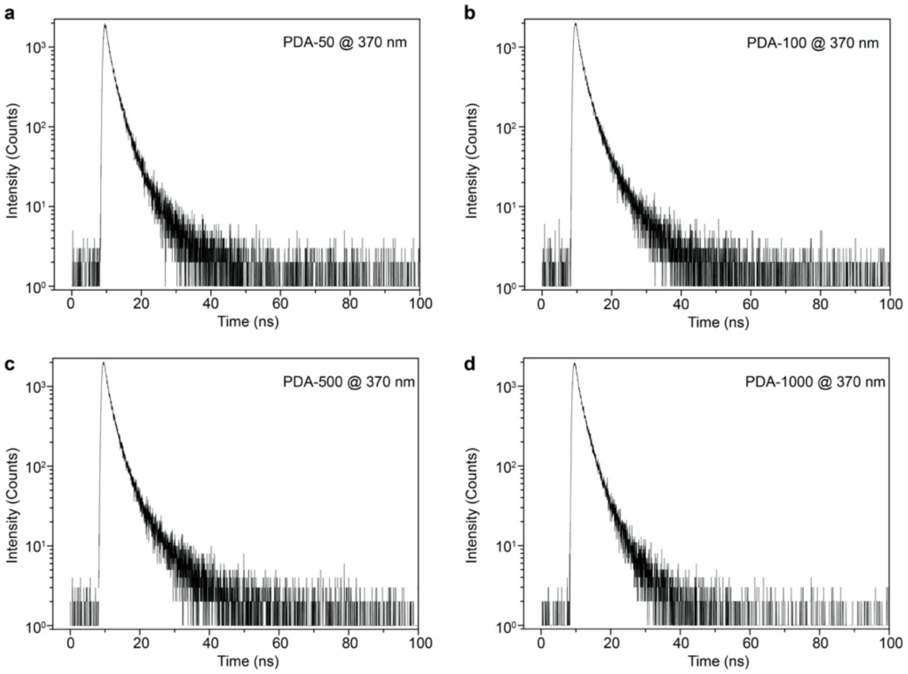

Supplementary Figure 28. **a-d**, Lifetime decay profiles of UOP band at 370 nm for PDA polymer film with different molar feed ratio of MDP : acrylic acid of 1:50 (PDA-50), 1:100 (PDA-100), 1:500 (PDA-500) and 1:1000 (PDA-1000) upon excitation at 300 nm under room temperature, respectively.

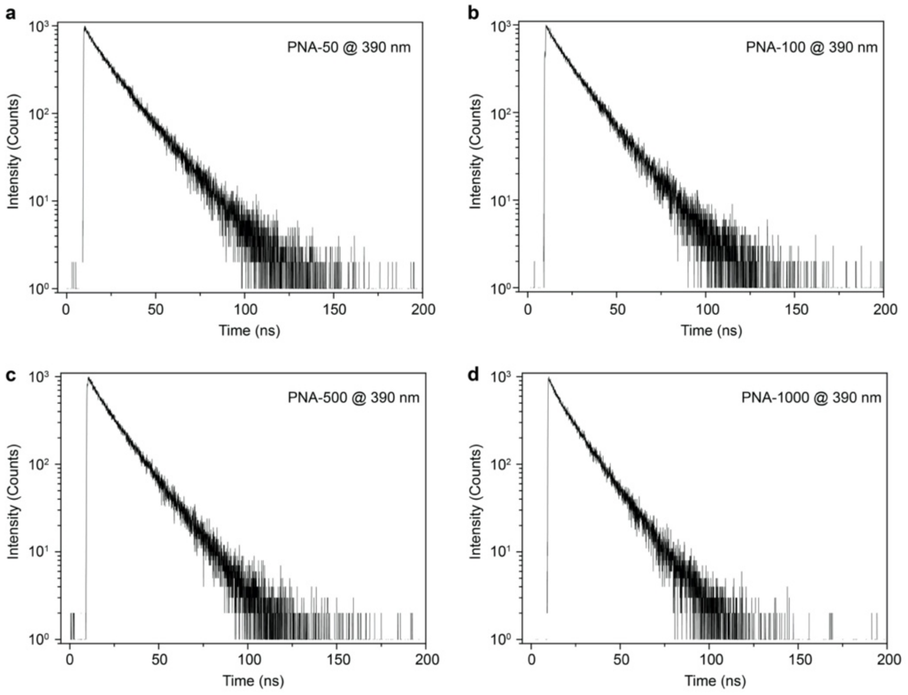

Supplementary Figure 29. **a-d**, Lifetime decay profiles of UOP band at 390 nm for PNA polymer film with different molar feed ratio of MND : acrylic acid of 1:50 (PNA-50), 1:100 (PNA-100), 1:500 (PNA-500) and 1:1000 (PNA-1000) upon excitation at 300 nm under room temperature, respectively.

Supplementary Table 4. Photoluminescence lifetimes (τ) of PDA and PNA films under ambient conditions.[a]

Compound	Wavelength (nm)	Fluorescence τ_1 (ns)	A_1 (%)	τ_2 (ns)	A_2 (%)	Phosphorescence τ_1 (ms)	A_1 (%)	τ_2 (ms)	A_2 (%)	τ_3 (ms)	A_3 (%)
PDA-50	370	1.35	52.99	3.96	47.01	-	-	-	-	-	-
	445					353.4	10.73	173.3	51.55	442.7	37.72
PDA-100	370	1.36	53.29	4.23	46.71						
	445	-	-	-	-	367.7	9.59	175.4	51.22	444.9	39.19
PDA-500	370	1.51	63.45	4.84	36.55	-	-	-	-	-	-
	445	-	-	-	-	365.6	8.13	200.8	47.54	520.6	44.32
PDA-1000	370	1.36	52.31	3.78	47.69						
	445	-	-	-	-	306.1	7.72	179.5	46.68	463.4	43.60
PNA-50	390	16.19	100	-	-	-	-	-	-	-	-
	517	-	-	-	-	156.0	20.54	555.7	79.46	-	-
	547	-	-	-	-	252.9	7.20	181.6	45.33	524.6	79.47
PNA-100	390	15.35	100	-	-	-	-	-	-	-	-
	517	-	-	-	-	413.6	2.47	381.9	33.51	987.6	64.02
	547	-	-	-	-	736.8	3.66	442.8	39.67	1,000	56.67
PNA-500	390	5.07	6.41	15.79	93.59	-	-	-	-	-	-
	517	-	-	-	-	263.5	1.59	388.7	50.73	776.9	47.68
	547	-	-	-	-	267.9	1.43	369.5	47.07	767.9	51.50
PNA-1000	390	3.98	7.07	15.09	92.93	-	-	-	-	-	-
	517	-	-	-	-	251.3	1.42	430.4	48.05	842.0	50.53
	547	-	-	-	-	146.1	0.64	406.1	44.26	835.0	55.11

[a]Determined from the fitting function of $I(t) = A_1^{e-t/\tau 1} + A_2^{e-t/\tau 2} + A_3^{e-t/\tau 3}$ according to the photoluminescence decay curves.

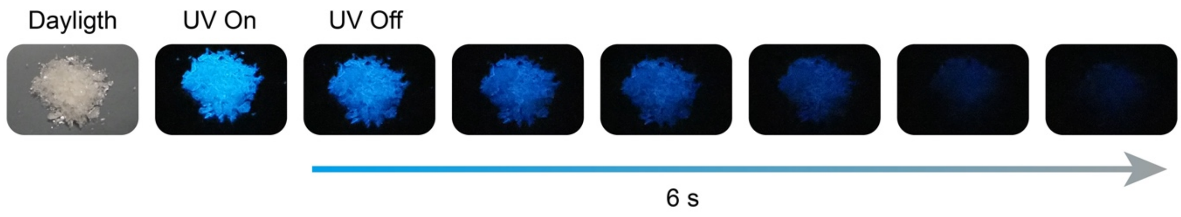

Supplementary Figure 30. Photographs of PDA polymer film monitoring the deactivation process of UOP excited at 254 nm under room temperature.

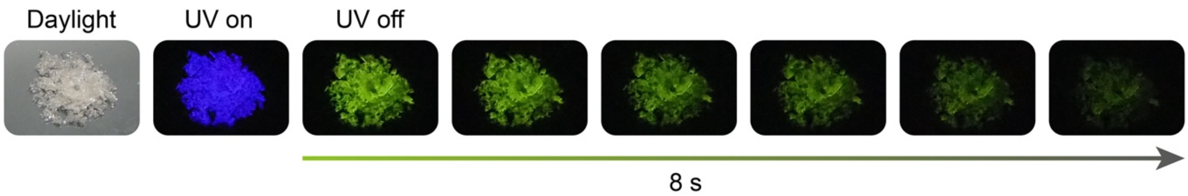

Supplementary Figure 31. Photographs of PNA polymer film monitoring the deactivation process of UOP excited at 365 nm under room temperature.

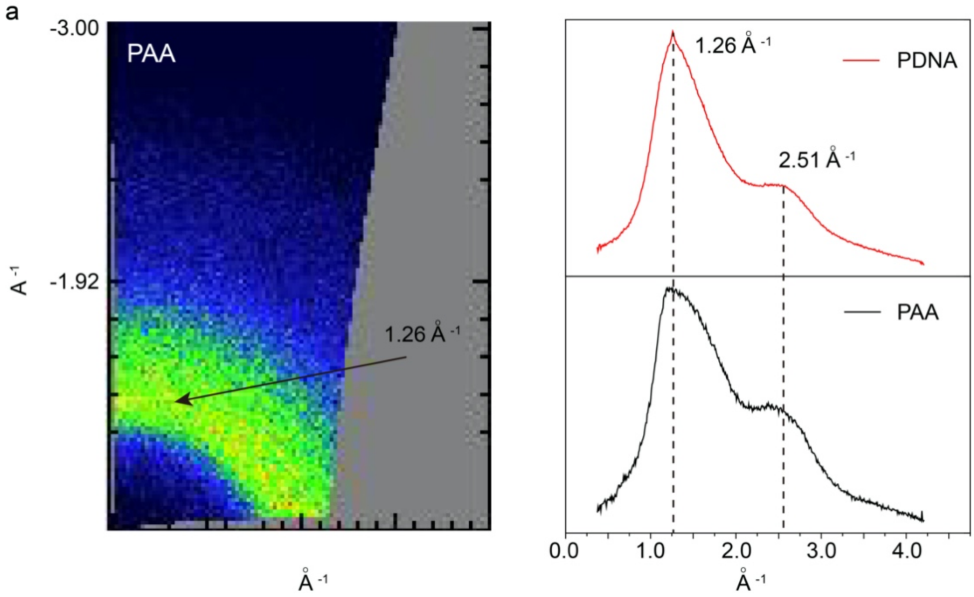

Supplementary Figure 32. Wide angle X-ray scattering pattern of polymer film PAA.

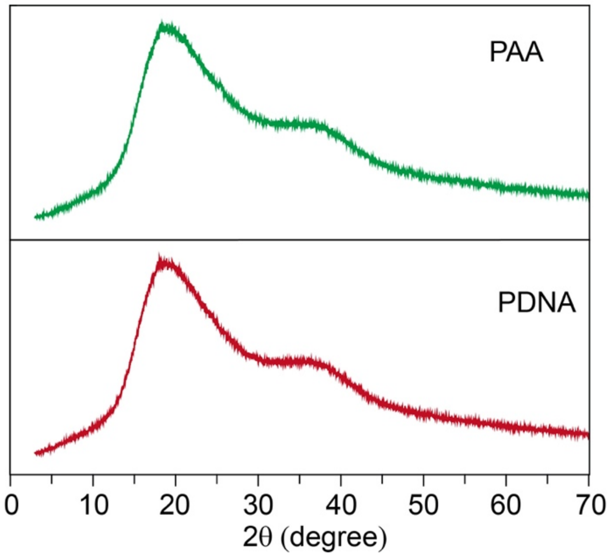

Supplementary Figure 33. Powder X-ray diffraction pattern of polymer films PAA and PDNA.

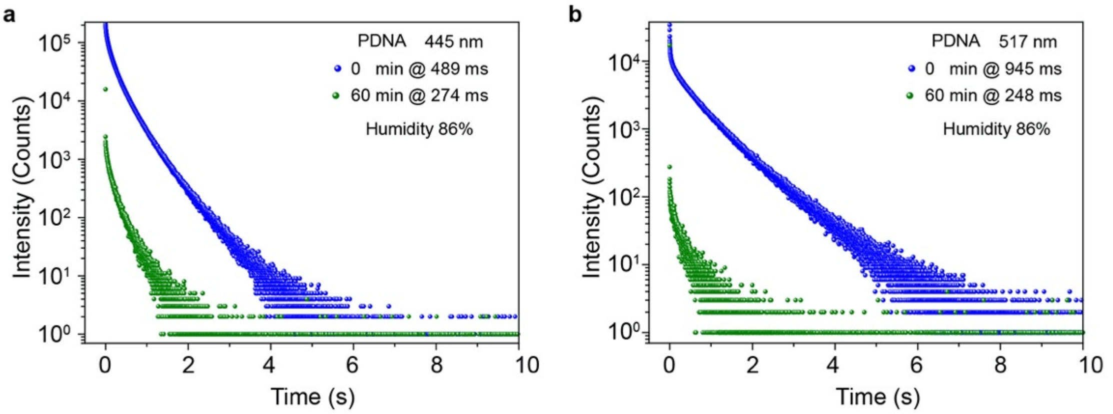

Supplementary Figure 34. **a**,**b**, Lifetime decay profiles of the emission bands around 445 and 517 nm for PDNA polymer exposed to wet air (the humidity is 86%) for 0 (blue line) and 60 (green line) minutes.

Supplementary Table 5. Phosphorescence lifetimes (τ) of PDNA film under humid environment (humidity is 86%).[a]

Compound	Wavelength (nm)	Phosphorescence							
		τ_1 (ms)	A_1 (%)	τ_2 (ms)	A_2 (%)	τ_3 (ms)	A_3 (%)	τ_4 (ms)	A_4 (%)
0 min	445	13.72	2.13	74.26	17.45	227.93	51.96	489.18	28.46
	517	29.28	1.28	143.05	6.06	497.28	49.99	945.53	42.68
60 min	445	64.69	32.06	274.78	67.94	-	-	-	-
	517	248.12	100	-	-	-	-	-	-

[a] Determined from the fitting function of $I(t) = A_1e^{-t/\tau 1} + A_2e^{-t/\tau 2} + A_3e^{-t/\tau 3} + A_4e^{-t/\tau 4}$ according to the phosphorescence lifetime decay curves.

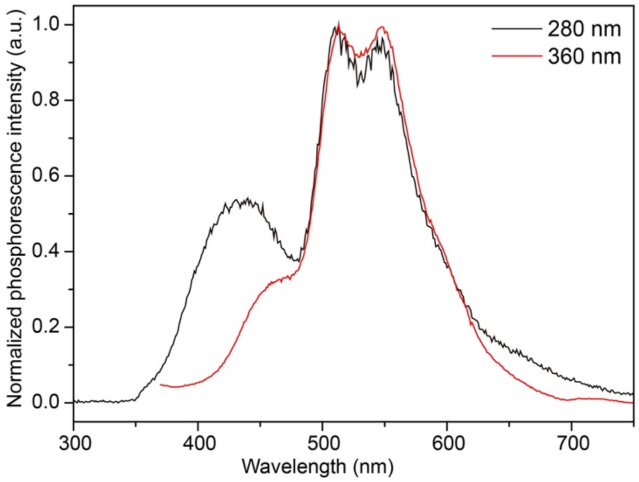

Supplementary Figure 35. Phosphorescence spectra of control polymer film excited at 280 (black line) and 360 nm (red line) under room temperature, respectively.

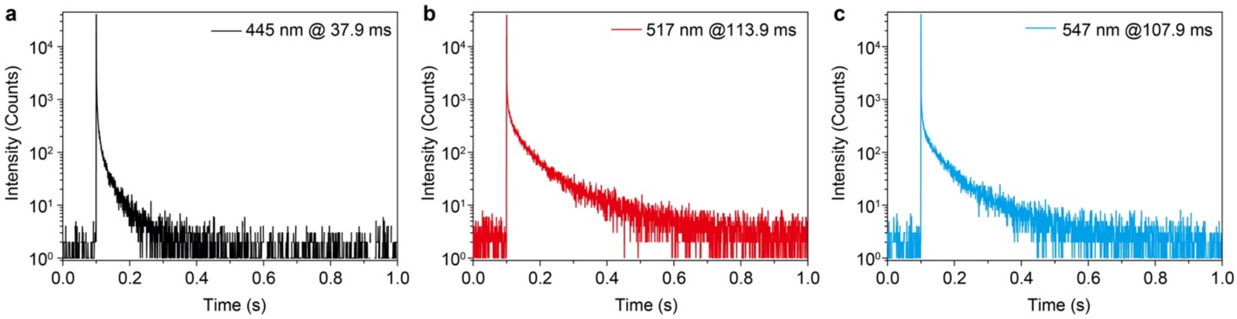

Supplementary Figure 36. **a-c**, Lifetime decay profiles of UOP bands at 445, 517 and 547 nm in control polymer film upon excitation at 280 and 360 nm under room temperature, respectively.

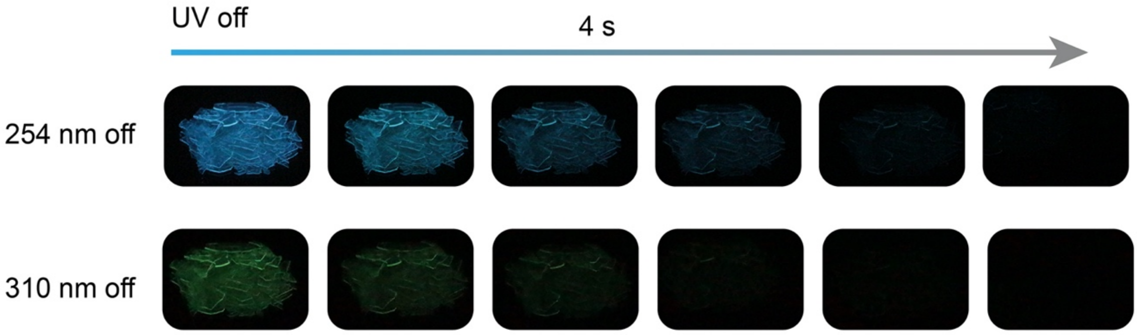

Supplementary Figure 37. Photographs of polymer film PDBA monitoring the deactivation process of UOP excited at 254 and 310 nm, respectively.

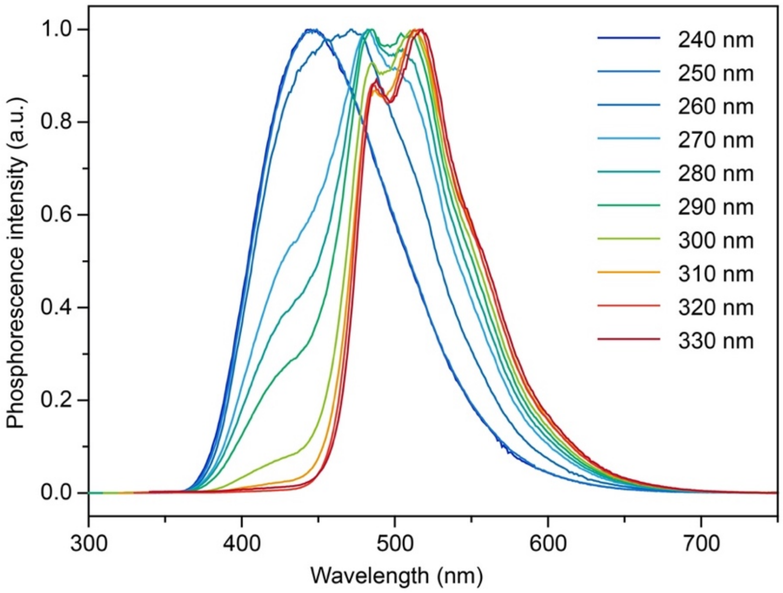

Supplementary Figure 38. Normalized phosphorescence spectra of PDBA polymer film upon excitation at 240, 250, 260, 270, 280, 290, 300, 310, 320 and 330 nm under ambient conditions.

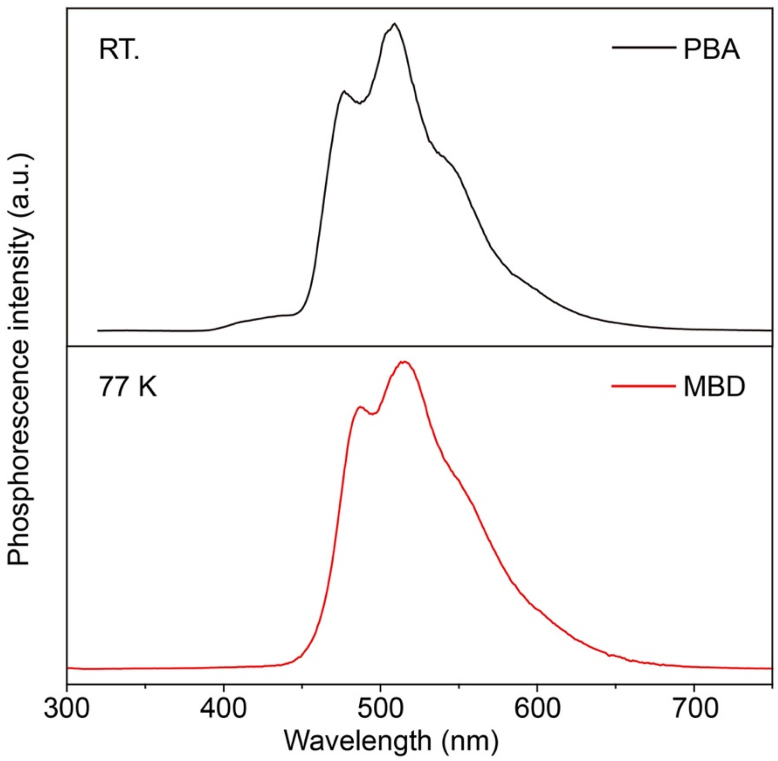

Supplementary Figure 39. Normalized phosphorescence spectra of polymer PBA at room temperature (black line) excited at 280nm and monomer MBD (red line) in 2-methyltetrahydrofuran (1×10^{-5} M) excited at 300 nm under 77 K.

Supplementary References

1. Ma, X., Xu, C., Wang, J. & Tian, H. Amorphous pure organic polymers for heavy-atom-free efficient room-temperature phosphorescence emission. *Angew. Chem. Int. Ed.* **57**, 10854-10858 (2018).
2. Zhang, L. et al. Potential anti-MDR agents based on the podophyllotoxin scaffold: synthesis and antiproliferative activity evaluation against chronic myeloid leukemia cells by activating MAPK signaling pathways. *RSC Adv.* **6**, 2895-2903 (2016).
