# Supplementary material for: Color-tunable ultralong organic room temperature phosphorescence from a multicomponent copolymer
Source: Nat Commun. 2020 Feb 18;11:944. doi: 10.1038/s41467-020-14792-1 (PMC7029031; doi:10.1038/s41467-020-14792-1)
Supplement: Supplementary file 2 — Description of Additional Supplementary Files [file 41467_2020_14792_MOESM2_ESM.pdf]

**Description of Additional Supplementary Files**

File Name: Supplementary Movie 1
Description: When excited with a 254 nm lamp, the PDNA sample emits a blue color. After switching-off of the lamp, sky-blue ultralong phosphorescence was clearly observed by the naked eyes. However, when changing the UV light from 254 to 365 nm, yellow persistent luminescence was visualized from the PDNA after the removal of the UV light.

File Name: Supplementary Movie 2
Description: This video demonstrates that when excited by a 254 nm lamp, the sample PDBA can emit visible blue ultralong phosphorescence. After turning off the excitation source of 310 nm, the transparent polymer film shows green long-lived emission.
